# Ultrahigh Density of Atomic CoFe-Electron Synergy in Noncontinuous Carbon Matrix for Highly Efficient Magnetic Wave Adsorption

**DOI:** 10.1007/s40820-022-00830-8

**Published:** 2022-04-06

**Authors:** Wenhuan Huang, Qiang Qiu, Xiufang Yang, Shouwei Zuo, Jianan Bai, Huabin Zhang, Ke Pei, Renchao Che

**Affiliations:** 1 Key Laboratory of Chemical Additives for China National Light Industry, College of Chemistry and Chemical Engineering, Shaanxi University of Science and Technology, Xi’an 710021, People’s Republic of China; 2 KAUST Catalysis Center, King Abdullah University of Science and Technology, 23955-6900 Thuwal, Kingdom of Saudi Arabia; 3 Laboratory of Advanced Materials, Shanghai Key Lab of Molecular Catalysis and Innovative Materials, Fudan University, Shanghai, 200438 People’s Republic of China

**Keywords:** Electromagnetic wave-absorbing materials, Off-axis electron hologram, M–M’ interaction, Hierarchical porous structure, Energetic metal organic framework

## Abstract

Improving the atom utilization of metals and clarifying the M–M’ interaction is both greatly significant in assembling high-performance ultra-light electromagnetic wave-absorbing materials. Herein, a high-temperature explosion strategy has been successfully applied to assemble the hierarchical porous carbon sponge with Co–Fe decoration via the pyrolysis of the energetic metal organic framework. The as-constructed hybrid displays a superior reflection loss (RL) value of − 57.7 dB and a specific RL value of − 192 dB mg^−1^ mm^−1^ at 12.08 GHz with a layer thickness of 2.0 mm (loading of 15 wt%). The off-axis electron hologram characterizes the highly distributed numerous polarized nanodomain variable capacitors, demonstrating the dipole and interfacial polarization along the edges of the nanopores. More importantly, the X-ray absorption spectroscopy analysis verifies the mutual interaction between the metal cluster and carbon matrix and the electronic coupling responsible for the greatly improved electromagnetic wave absorption.

**Supplementary Information:**

The online version contains supplementary material available at 10.1007/s40820-022-00830-8.

## Highlights

- A typical 3D porous carbon sponge of CoFe@PCS exhibited the continuous distribution of nano-meso-micro-hierarchical pores in the range of 1 nm–15 μm.
- The ultrahigh-density distribution of the nanoscale polarized charges (+ / −) along the edges of the pores resulted in nanoscale variable capacitors.
- The high density of Co–Fe electromagnetic coupling on the carbon matrix, showing the enhanced electromagnetic wave attenuation.

**Supplementary Information:**

The online version contains supplementary material available at 10.1007/s40820-022-00830-8.

## Graphical abstract

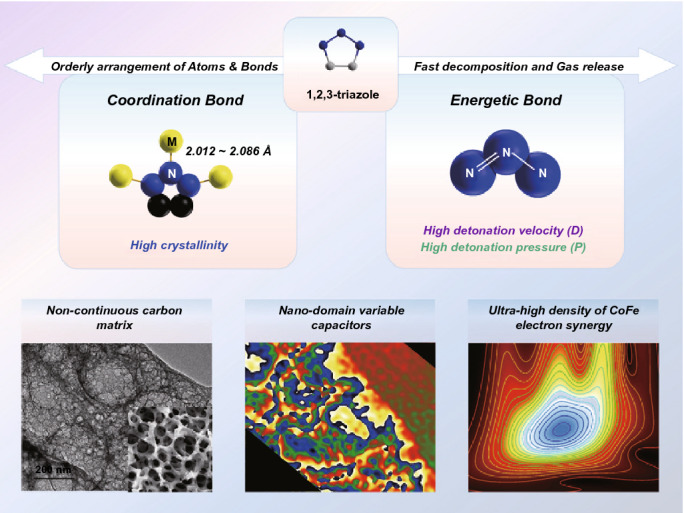

## Introduction

With the rapid development of 5G technology, numerous electronic devices that rely on electromagnetic waves (EWs) as information carriers have been widely applied in various fields [1–5]. Despite their significant contributions to global communication, these equipment and devices also bring severe radiation pollution [6–11]. Hence, high-performance EW-absorbing and shielding materials [12–16] have emerged as research hotspots, showing promising applications in civilian and military fields, such as radiation protection [17–20] and military stealth coatings [21–26].

Recently, a wide variety of electromagnetic wave-absorbing materials (EWAMs) have been exploited [27], including carbon-based nonmetallic materials [28–30], polymers [23, 31, 32], metallic carbides [33–37], oxides [38–41], nitride [42], sulfides [43–45], and their composites. Among them, magnetic metal/carbon composites combine both the merits of the high conductivity of the carbon matrix and the excellent magnetism of metallic compounds, displaying superb EW-absorbing performance through the great balance between dielectric loss and magnetic loss [46–49].

In designing and synthesizing this kind of EWAM, selecting the metals and controlling their proportion, distribution, and existence form in the carbon matrix are critical to achieve high EW absorption [50]. In particular, to satisfy the high demands of thinness, lightweight, and low cost, many strategies have been employed to decrease the metal proportion without sacrificing the EW-absorbing performance [51, 52]. In addition, reducing the size of metallic particles and embedding them in a highly homogeneous matrix can effectively prevent aggregation [53–55].

Some successful approaches have been demonstrated in recent years. For example, a cage-confinement strategy has successfully assembled tiny MnO_2_ nanoparticles into two-dimensional (2D) support [56, 57]. Moreover, cycled annealing treatment on embedded Sn nanocrystals realized the multisplitting of nanoparticles, obtaining a size reduction and phase conversion [58]. In the future, simple and facile synthesis approaches of these fine nanoscale materials are crucial to promoting their industrial applications, which deserve further investigation [59]. Besides, the hierarchical porous nanomaterials generally result in great balance of electromagnetic wave reflection and adsorption through the air-filled nano-/meso-free spaces which could be obtained through the bottom-to-up way by employing hard porous template and the top-to-down method by using energetic precursors [60].

In addition, introducing multiple types instead of a single kind of metal into a composite could enhance the magnetic loss through the electromagnetic coupling effect between different metals, which is also an efficient way to enhance the EW attenuation performance [61, 62]. Thus far, many CoFe- or CoNi-bimetallic nanomaterials with special morphologies have exhibited highly improved EW-absorbing performance [63–66]. Additionally, the important polarization and electromagnetic synergistic effect among the interfaces of various magnetic particles in these materials has been proved and instrumentally observed by off-axis electron hologram and charge density map [67, 68]. However, the objects involved in these studies remain at the nanoscale, such as nanoparticles or nanointerfaces. The unclear arrangements and locations of metal sites in these materials create many barriers in hindering the investigation of the M–M’ interaction and designing bimetallic EWAMs at the atomic level [69]. Hence, a suitable material platform with highly distributed atomic metal sites must be constructed, which is helpful in unveiling the coupling mechanism of the M–M’ interaction.

**Scheme 1:**
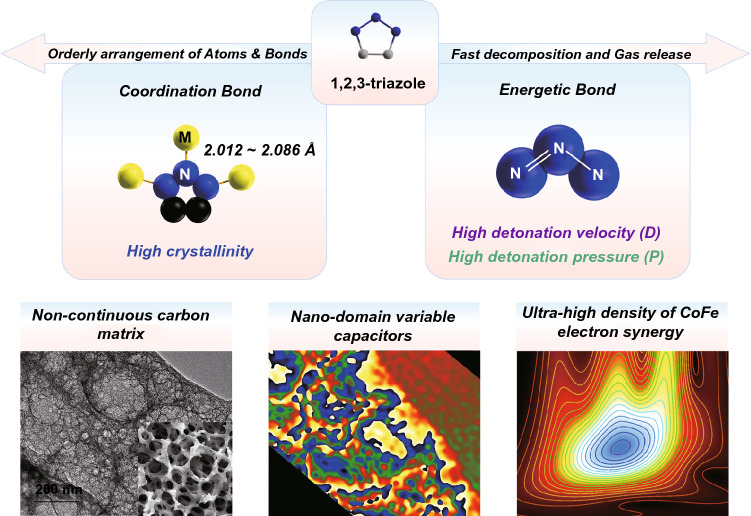
Material design strategy and characteristics of CoFe@PCS

Herein, by employing 1,2,3-triazole (with N–N = N bond) as a ligand, a high-crystalline energetic metal organic framework (MOF) with low CoFe proportion was synthesized as a precursor (CoFe@MET) to construct highly efficient EWAMs (Scheme 1). Owing to the energetic N_3_-bond in the precursor, CoFe@MET was in situ transformed into an atomic CoFe-doped atypical 3D porous carbon sponge (CoFe@PCS) during the high-temperature explosion process. The characterizations revealed the continuous distribution of hierarchical pores in the range of 1 nm–15 μm in the carbon matrix, providing an ideal platform for the homogenous dispersion of atomic Co and Fe (~ 0.6 wt%) sites and the related Co-Fe interactions. The off-axis electron holography demonstrated the polarization and electron coupling along the nanopores in CoFe@PCS, greatly promoting EW absorption. At a low loading of 15 wt%, CoFe@PCS displayed a high reflection loss (*RL)* value of − 57.7 dB and a specific *RL* value (S_*RL*_*)* of − 192 dB mg^−1^ mm^−1^ at 12.08 GHz under the layer thickness of 2.0 mm. More importantly, the extended X-ray absorption fine structure (EXAFS) and X-ray absorption near-edge structure (XANES) revealed the coordination and bonding information around the Co and Fe atoms on the carbon matrix, presenting an excellent example of the atomic-scale structure design.

## Experimental Section

### Synthesis of CoFe@MET Precursor

The ZnCl_2_ (5.0 g) was dissolved in a mixture of ethanol (50 mL), water (75 mL), ammonium hydroxide (25–28%, 20 mL), and N, N-dimethylformamide (50 mL), stirring for 10 min. Afterward, 1H-1,2,3-triazole (6.26 mL) was slowly dropped into the solution during stirring. After 24 h of stirring at room temperature, the white product of MET was generated and filtered. Then, the MET (2.0 g) powder was immersed into a solution of Co(CH_3_COO)_2_·6H_2_O (0.72 g), FeCl_2_ (0.49 g), and methanol (200 mL), stirring at room temperature for 6 h. The light pink powder was filtered and washed using ethanol three times. After vacuum drying at 60 °C for 8 h, CoFe@MET was collected with a yield of 62%. In contrast, the Fe@MET and Co@MET precursors were synthesized, and the synthesis details are listed in supporting information.

### Synthesis of CoFe@PCS

The as-prepared 2.0 g CoFe@MET was placed into a ceramic boat and then the programmed tube furnace. It was heated to 900 °C at a heating rate of 5 °C min^−1^ under a nitrogen atmosphere. Afterward, the furnace was maintained at 900 °C for 2 h and then naturally cooled to room temperature. The ultra-light black powder of CoFe@PCS (0.16 g) was successfully synthesized. Similarly, Fe@PCS and Co@PCS were synthesized (details in supporting information).

### Characterizations

A D8 DaVinci X-ray powder diffractometer (XRD) equipped with graphite-monochromatized Cu Kα radiation (λ = 0.1542 nm) was used to record the XRD patterns in the 2θ range of 5–80° with a scanning rate of 1° min^−1^. The Brunauer–Emmett–Teller method calculated the specific surface area through nitrogen adsorption and desorption at 77 K using the ASAP 2020 sorption system. The scanning electron microscopy (SEM) images were collected using a Hitachi S4800 apparatus with an acceleration voltage of 2 kV. The transmission electron microscopy (TEM) images were recorded on JEM-2100F, JEM-2010HR, and FEI Talos F200X, working at an accelerating voltage of 200 kV, and the X-ray energy-dispersive spectroscopy was taken on a JEM-2010HR-Vantage-type energy spectrometer. The XPS was implemented on a Thermo ESCA Lab250XI. The Raman spectroscopy of the samples was obtained using a Renishaw via a Raman microscope. The electromagnetic parameters were analyzed using an HP8753D vector network analyzer in the frequency range of 2–18 GHz. The measured samples were homogeneously dispersed in paraffin with a sample-to-paraffin weight ratio of 3:17, and the mixture was pressed into a toroidal shape with an inner diameter of 2.0 mm and an outer diameter of 7.0 mm. The conductivity of the samples (1 × 1 cm^2^) was performed through an ST2253 four-probe resistance meter. The hysteresis loop of the materials was tested using a superconducting quantum interference device MPMS (SQUID) VSM magnetometer. The absorption spectra of the Mo-edge were collected in transmission mode at room temperature using an Si (111) double-crystal monochromator at the 1W1B station of the Beijing Synchrotron Radiation Facility.

### Data Analysis

The reflection loss (*RL*) value of the absorber was calculated according to the transmission line theory. The polarization process was calculated using the Cole–Cole semicircle model. Polarization relaxation and charge transport in the dielectric loss were calculated using the Debye relaxation correction formula. The calculation details are listed in Supporting Informations.

## Results and Discussion

### Construction and Characterization of CoFe@PCS

**Fig. 1:**
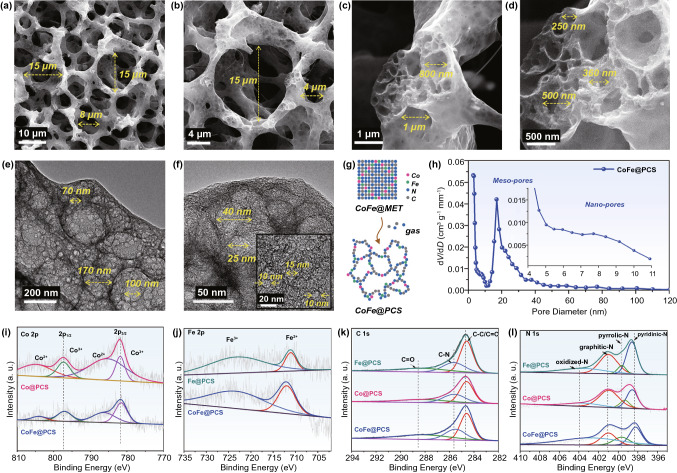
Porous characterization of the 3D noncontinuous carbon matrix in CoFe@PCS. **a-d** SEM images with the pores of 200 nm–15 μm. **e–f** TEM images with pores of 10–200 nm. **g** Scheme of formation of the pores in the CoFe@PCS. **h** Pore diameter distribution curves. **i-l** The XPS spectra of Co 2*p* (**i**), Fe 2*p* (**j**), C 1* s* (**k**), N 1* s* (**l**) for Fe@PCS, Co@PCS, CoFe@PCS

The synthesis procedure of the CoFe-embedded porous carbon sponge (CoFe@PCS) is illustrated in Fig. S1. Firstly, an energetic MOF (MET) crystallized from Zn and 1,2,3-triazole (N_3_) was immersed in a Co^2+^ and Fe^2+^ solution for 6 h and was filtered out as precursor (CoFe@MET). In addition, CoFe@MET inherited the original morphology of MET, displaying an octahedron with an average diameter of 100 nm (Fig. S2). The Co and Fe atoms were doped on the surface of the CoFe@MET crystals without changing the crystalline structure, which was confirmed via TEM (Fig. S6) and XRD investigation (Fig. S7). During the calcination process at the range of room temperature to 900 °C under nitrogen flow, the unstable N–N = N bonds in 1,2,3-triazole exploded at ~ 440 °C (Fig. S9), whereas the Co and Fe atoms were highly dispersed within the carbon matrix (CoFe@PCS) during the gas departure and volume expansion process (Fig. 1g). The point of the explosion and the decomposed temperature were evaluated using DSC and TGA.

After heat treatment, the precursor was in situ transformed into the hierarchical carbon sponge, which revealed an ultralow density (Fig. S18) and high porosity (Fig. 1). The SEM and TEM results (Fig. 1a–f) indicate that the diameters of the pores are continuously distributed within a wide range of 10 nm–15 μm, which was also demonstrated by the pore size distribution map fitted by N_2_ adsorption–desorption isotherms (Fig. 1h). Such a hierarchical porous structure of CoFe@PCS provided an atypical 3D noncontinuous carbon matrix unlike other carbon composites, which facilitates high dispersion and efficient utilization of metals sites. More importantly, it may provide a great opportunity for generating atomic metal sites and studying the existing status of the M-M’ units.

### Electromagnetic Wave-Adsorbing Performance

**Fig. 2:**
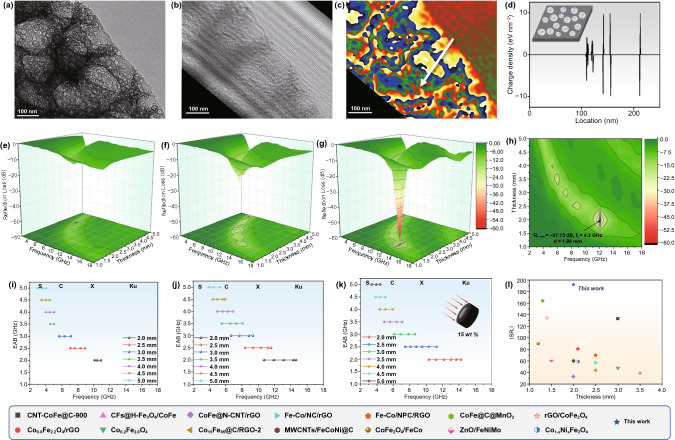
Nanodomain variable capacitors and electromagnetic wave (EW) attenuation performance. **a‑b** HR-TEM and corresponding hologram images. **c** Charge density map. **d** Charge density profile along the white arrow and scheme of nanodomain variable capacitors (insert). **e‑g** The electromagnetic parameters, 3D *RL* values of Fe@PCS, Co@PCS, and CoFe@PCS. **h** 2D *RL* projection of CoFe@PCS. **i-k** Effective absorption bandwidth (< –10 dB) of Fe@PCS, Co@PCS, and CoFe@PCS. **l** EW-absorbing performance comparison of CoFe@PCS with other CoFe-based materials (see Table S2 in supporting information)

In CoFe@PCS, a small number of Co and Fe atoms (0.344 and 0.316 wt%, respectively) were inserted on the carbon surface in a highly dispersed manner. Owing to the high specific surface of CoFe@PCS, the doped metal sites in the carbon matrix brought a high density of dipole polarized units, which reflected in the nanoscale are abundant nanodomain variable capacitors. As confirmed by off-axis electron holograms and the corresponding charge density map (Fig. 2a–c), the high distribution of charge polarization spaces is highly matched with the distribution of nanopores. Along with the white arrow in Fig. 2c, the polarized positive and negative charges (3 to 10 eV) are distributed on edges at both sides of the pores (~ 10 nm; Fig. 2d).

Generally, the loading amount of filler in a testing ring is in the range of 30–50% to achieve high EW adsorption. However, owing to the highly distributed atomic metal sites and ultralow density of CoFe@PCS, a small loading of 15 wt% was applied in this work. The reflection loss (*RL*) and |Zin/Z0| of the CoFe@PCS were calculated from the tested electromagnetic parameters through Eqs. S1–S2 (Fig. 2e–g). To our delight, a high RL value of –57.73 dB at 12.1 GHz (Fig. 2e) and a wide effective adsorption band (<  − 10 dB) of 4.2 GHz (Fig. 2g) at the thickness of 2.0 nm were observed, indicative of the excellent EM absorption performance. For further comparison with other reported CoFe-based materials, the SRL value, which considers the loading and layer thickness of the test ring, was calculated according to Eq. S3 (Fig. 2l). In addition, CoFe@PCS displays a remarkable *SR*_*L*_ of − 192 dB mg^−1^ mm^−1^, which is far beyond the values for CoFe-based materials reported previously (Table S2). Moreover, unlike most metal/carbon composite EWAMs, the ultralow density and metal proportion (only around at ~ 0.6 wt%) make it a promising light-weight EWAM.

Single-metallic Fe- and Co-embedded porous carbon sponges (Fe@PCS and Co@PCS) were synthesized as contrast samples to investigate the EW attenuation mechanism (Fig. S1). The electron microscope characterization of Fe@MET and Co@MET precursors revealed similar octahedron morphology, particle size (Figs. S2–S6), and crystalline structure (Fig. S7) with CoFe@MET, while the Fe@PCS and Co@PCS after calcination also displayed the same hierarchical porous structures as CoFe@PCS (Figs. S10–S16). Generally, the Co and Fe atoms in the precursors display different catalytic abilities in transforming graphitic carbon in the composite during high-temperature treatment. However, according to the PXRD and Raman results (Fig. S17), the scarce metal content in these three precursors resulted in a negligible difference in the *I*_D_/*I*_G_ ratios (0.96–1.03) in the final composites. Hence, the M@PCS in this work provides an ideal platform for evaluating the M-M’ interactions in the EW attenuation process due to the consistent nanostructures, except for metal sites.

The *RL*, 3D *RL*, and |*Z*_in_/*Z*_0_| values of three M@PCS samples were calculated and are illustrated in Figs. 2e–k and S18–S22. A higher *RL* value of CoFe@PCS (− 57.73 dB at 12.08 GHz) than the values of Co@PCS and Fe@PCS (− 29.68 dB at 6.64 and − 14.72 dB at 3.92 GHz) and a wider effective bandwidth (*f*_e_, frequency range of *RL* value below − 10 dB) of CoFe@PCS (4.2 GHz) than the values of Co@PCS and Fe@PCS (2.48 and 0.95) were observed, indicating the superior EW attenuation of CoFe@PCS. These results demonstrate the key role of the M-M’ interaction in CoFe@PCS.

**Fig. 3:**
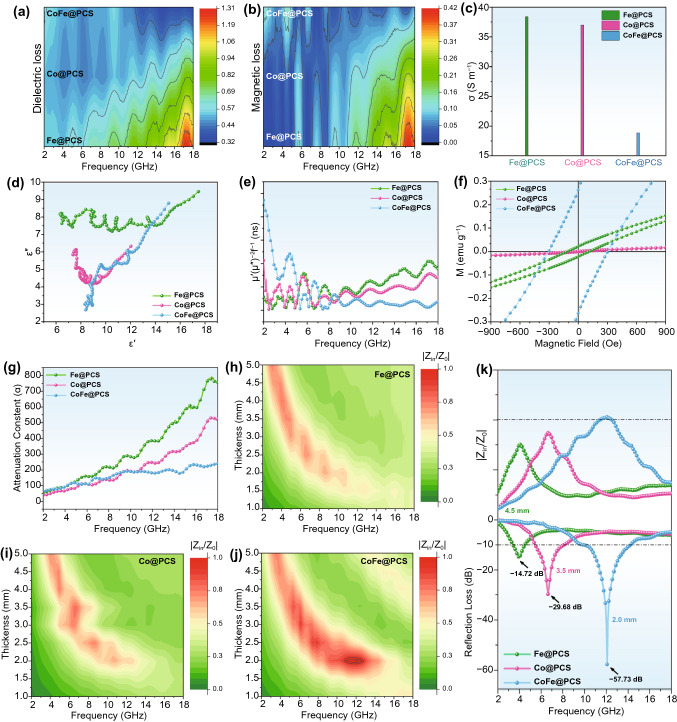
EW attenuation and impedance matching analysis. **a** 2D contour map of tan *δ*_ε_ for dielectric loss. **b** 2D contour map of tan *δ*_μ_ for magnetic loss. **c** Attenuation constant. **d** Electrical conductivity. **e** Cole–Cole semicircle. **f** *C*_0_ value. **g** Magnetic hysteresis loop. **h-j** 2D contour maps of |*Z*_in_/*Z*_0_|. **k** Best *R*_*L*_ values and corresponding |*Z*_in_/*Z*_0_| for Fe@PCS, Co@PCS, and CoFe@PCS

Therefore, the EM parameters calculation and relevant characterization of three M@PCS materials were conducted and analyzed to study the role of the M-M’ interaction in CoFe@PCS during the EW-absorbing process. The attenuation constant (*α*), calculated using Eq. S4, is a parameter representing the attenuation ability of the materials, consisting of dielectric loss and magnetic loss. As illustrated in Figs. 3 and S21, relatively stronger permittivity and permeability of Fe@PCS than those of CoFe@PCS and Co@PCS were observed in the dielectric and magnetic loss 2D contour maps (Fig. 3a,b), which are fitted from tan *δ*_ε_ and tan *δ*_μ_ values. These results were highly agreed with the attenuation constant (Fig. 3c). The real part of the permittivity and permeability exhibited higher *ε*′ and *µ*′ values for CoFe@PCS than Co@PCS. However, the higher *ε*′′ and *µ*′′ values of Co@PCS than CoFe@PCS at a high-frequency range (> 10 GHz) resulted in the corresponding higher attenuation constant of Co@PCS (Fig. S21).

Dielectric loss is the main attenuation in carbon-based nanomaterials, containing conductive and polarization contributions. Firstly, the electrical conductivity (σ) of three materials was tested using a four-probe resistance meter (Fig. 3d), displaying the highly conductive contribution of the single-metallic Fe@PCS and Co@PCS. Moreover, the polarization relaxation process of the materials was studied through the Cole–Cole semicircle mode (Eq. S5), and the contribution of polarization relaxation and the charge transport (${\varepsilon }_{p}^{\mathrm{^{\prime}}\mathrm{^{\prime}}}$ and ${\varepsilon }_{c}^{\mathrm{^{\prime}}\mathrm{^{\prime}}}$) in the dielectric loss was calculated according to the Debye theory (Eqs. S6-S10). Figures 3e and S22 present a relatively stronger polarization of Fe@PCS, compared with Co@PCS and CoFe@PCS. However, the polarization maps and values of these materials are very close, which could be further experimentally confirmed using off-axis electron holograms and charge density maps. Figures 2a–d and S23–S24 reveal three materials displaying strong interfacial polarization. In such a hierarchical porous structure, the polarization loss of the materials is primarily derived from the nonporous structures and chemical bond dipoles, which indicates the Co-Fe interaction may play key roles in magnetic loss and impedance matching.

As another part of the attenuation contribution, the magnetic loss of the materials can be evaluated by the eddy current loss (*C*_0_) through Eq. S11. Figure 3f presents higher *C*_0_ values for CoFe@PCS than Co@PCS and Fe@PCS in the low-frequency region (< 7 GHz); however, the *C*_0_ values of CoFe@PCS decrease gradually with increasing frequency in an opposite tendency with Co@PCS and Fe@PCS. However, owing to the low metal proportion in these three materials, the calculated *C*_0_ values are very low only around 0–0.02 ns. Moreover, the fitted magnetic hysteresis loops of the materials (Figs. 3g and S25) clearly exhibit the highest saturation magnetization (*M*s) and coercive force (*H*c) for CoFe@PCS among three samples, confirming the electromagnetic coupling between the Co and Fe atoms. Therefore, we can conclude that in the CoFe@PCS, the dielectric loss is the dominant contribution of the EW attenuation, which is determined by the conductivity of the carbon matrix and the chemical dipole and defects on the surface of the hierarchical porous structure. Although the magnetic loss is not the leading contribution in EW attenuation, the strongest magnetic loss of the CoFe@PCS resulting from the Co–Fe coupling is observed.

Furthermore, the impedance matching of three materials was analyzed. Impedance matching directly determines the EW absorbing ability by balancing the impedance between the substance and free space. Generally, designing the morphology of the materials with suitable hollow or porous micro-nanostructures can effectively control impedance matching. However, the chemical composition and atomic electronic structure influences in materials are still unclear. In this work, the same porous morphology and highly distributed atomic sites in three materials offer an excellent chance for investigating the key role of the Co-Fe interaction on impedance matching. The impedance matching of three materials (Fig. S26) was calculated using the normalized characteristic impedance (*Z* =|*Z*_in_/*Z*_0_|) based on Eq. S2. As presented in Fig. 3h–j, the red area (*Z* = 0.8–1.1) in the 2D contour maps of the *Z* values indicates good impedance matching. Compared with the single-metallic Co@PCS and Fe@PCS, the great matching of impedance for CoFe@PCS (close to 1) directly resulted from its superior EW absorption (Fig. 3k). Owing to the hierarchical porous structure of CoFe@PCS, the air-filled nano- and mesopores greatly prevent the reflection in the matrix and promote the adsorption; moreover, the meso- and micro-scale pores enhanced the multi-reflection of the escaped EW. However, the best matching of impedance for CoFe@PCS indicated that the chemical M-M’ dipole in the nanoporous matrix/air greatly enhances the EW adsorption. To the best of our knowledge, the key role of the atomic Co–Fe interaction in enhancing impedance matching of the material was first proposed in this work.

### M-M’ Interaction Evaluation in EW Adsorption

**Fig. 4:**
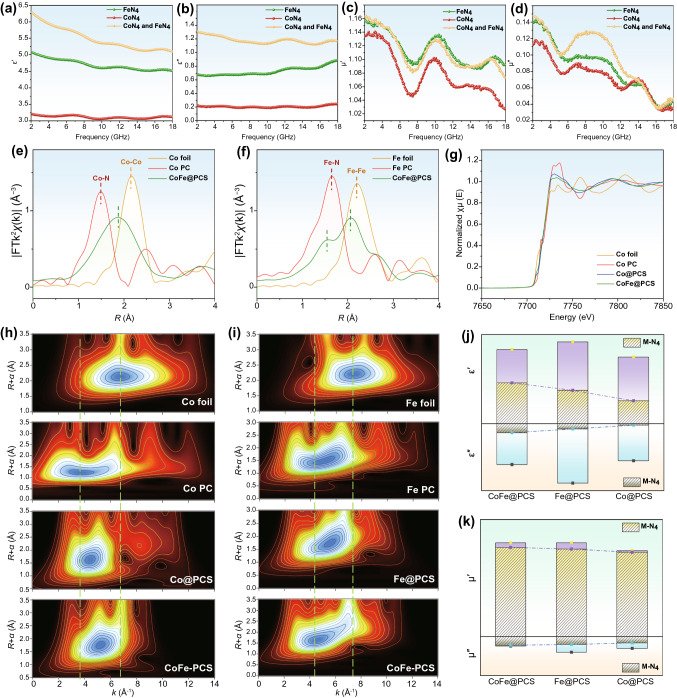
Analyses of Co-Fe interaction in CoFe@PCS. **a** The real part and **b** imaginary part of permittivity. **c** Real part and **d**) imaginary part of permeability for Fe–N_4_, Co–N_4_, CoFe–N_4_ in Fe@PCS, Co@PCS, and CoFe@PCS. **e** K-edge EXAFS spectra for Co foil, Co PC, and CoFe@PCS. **f** K-edge EXAFS spectra for Fe foil, Fe PC, and CoFe@PCS. **g** Experimental XANES spectra. **h-i** Wavelet transform for the *k*^2^-weighted EXAFS signals. **j-k** The permittivity and permeability of Fe–N_4_, Co–N_4_, and CoFe–N_4_ in Fe@PCS, Co@PCS, and CoFe@PCS

The extended EXAFS spectroscopy was applied to clarify the three samples’ local atomic and electronic structures to further study the atomic Co–Fe interaction. The Fourier transform of the Co K-edge EXAFS (Fig. 4e) for Co foil, cobalt(II) phthalocyanine (Co PC), and CoFe@PCS indicated that the bond distance around the Co atom (1.86 Å) in CoFe@PCS lies between Co–N (1.49 Å) and Co–Co (2.17 Å) bonds. The Fourier transform of the Fe K-edge EXAFS (Fig. 4f) for CoFe@PCS compared with Fe foil and iron (II) phthalocyanine (Fe PC) revealed the coexistence of isolated Fe atoms and Fe–Fe/Co interaction. The bonding between the Co and Fe atoms changes their coordination environments in CoFe@PCS, displaying the shifts of the bond lengths. The X-ray absorption near-edge structure (XANES) results (Fig. 4g) were performed to identify the valence state of the metal sites. The valence of Co in CoFe@PCS lies between the Co foil and Co@PCS, indicating the charge transfer from Fe to Co, resulting in the valence rise of Co in CoFe@PCS. In Fig. 4h,i wavelet transform simulation images of Co and Fe displayed the visualized radial distance resolutions in K space. The Co and Fe radial distances indicate a positive shift of Co and negative shift of Fe in CoFe@PCS, compared with the single-metallic Co@PCS and Fe@PCS. These accurate chemical characterization results reveal the chemical bonding and electron transfer between Co and Fe sites, which agrees with the X-ray photoelectron spectroscopy (XPS) results (Fig. 1i–l).

Furthermore, the EW-absorbing performance of the three HF-treated samples was measured to compare and evaluate the contribution of these Co and Fe sites (Co–N_4_ and Fe–N_4_) in the carbon matrix (Figs. 4a–d and S27–S29). As presented in Fig. 4j,k, Co/Fe–N_4_ in CoFe@PCS displayed the strongest permittivity and permeability contribution (*ε*′, *ε*′′, *µ*′, and *µ*′′) among the three materials, revealing the sequence CoFe@PCS > Fe@PCS > Co@PCS. These results indicate that the coordination among Co and Fe realizes the charge transfer from Fe to Co, changing the existing state of the metal sites and further enhancing the EW attenuation in CoFe@PCS.

### Electromagnetic Wave Adsorption Mechanism

**Fig. 5:**
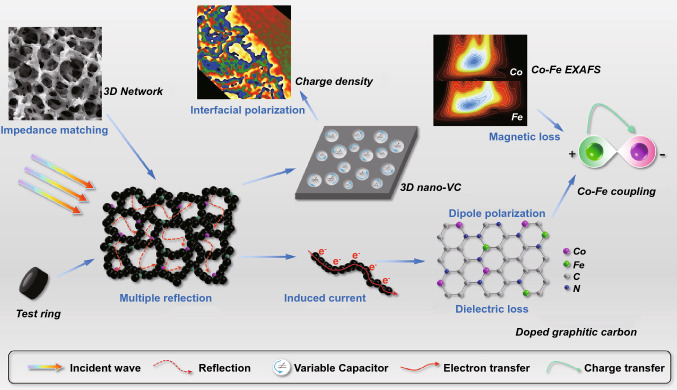
Schematic diagram of the electromagnetic wave adsorption mechanism for CoFe@PCS

At last, the EW absorbing mechanism of CoFe@PCS, a unique Co-Fe coupling un-continuous hierarchical porous carbon network, is illustrated as Fig. 5. The continuous high-density distribution of nano-meso-micropores at the range of 1 nm–15 μm resulted in the synergistic enhancement of the multi-reflection though the micro-scale pores and impeding matching through the air-filled nano- and mesospaces, displaying the optimal EW adsorption [70]. The electron transfer on the graphic carbon induced electron current on the matrix. In addition, although only ~ 0.6 wt% atomic Co and Fe were loaded on the carbon sponge matrix in CoFe@PCS, the atomic-doped Co–Fe heteroatoms and defects on the graphic carbon resulted in the high density of chemical dipoles. The Co/Fe, metals/carbon, and matrix/air in the structure provide abundant heterointerfaces for interfacial polarizations, giving numerous nanoscale variable capacitors. The Co–Fe electromagnetic coupling induced the intense magnetic loss of the materials. Combining the intense dielectric and magnetic loss of the CoFe@PCS, the promoted attenuation and impendence matching displaying the optimal EW absorbing performance.

## Conclusion

Improving the atom utilization of the metals and investigating the M-M’ interaction is of great significance for assembling high-performance ultra-light EWAMs. In this work, a CoFe-soaked energetic MOF with N–N = N bonds as a precursor successfully constructed an atypical 3D porous carbon sponge (CoFe@PCS), exhibiting the continuous distribution of hierarchical pores in the range of 1 nm–15 μm. In CoFe@PCS, only ~ 0.6 wt% atomic Co and Fe were homogeneously dispersed on the carbon sponge matrix (CoFe@PCS). Owing to the low density and metal proportion, CoFe@PCS (loading of 15 wt%) displayed a superior EW-absorbing performance of *R*_*L*_ of − 57.7 dB and *SRL* of − 192 dB mg^−1^ mm^−1^ at 12.08 GHz under the layer thickness of 2 mm.

Benefit from the 3D conductive network, the doped Co/Fe atoms and defects on the graphic carbon further delivered the high density of chemical dipoles, resulting in numerous nanoscale variable capacitors. The off-axis electron holography and charge density maps experimentally confirmed the ultrahigh-density distribution of the nanoscale polarized charges (+ / −) along the edges of the pores in CoFe@PCS. Furthermore, the atomic Co-Fe interaction was investigated by EXAFS and XANES, revealing that the chemical bonding between Co and Fe realizes the charge transfer from Fe to Co and further enhances the EW adsorption with the great balance of EW attenuation and impedance matching. This work presents an excellent example of the atomic-scale structure design of EWAMs.

## Supplementary Information

Below is the link to the electronic supplementary material.

Supplementary file1 (PDF 3617 KB)
