# Supplementary material for: Ultrahigh Density of Atomic CoFe-Electron Synergy in Noncontinuous Carbon Matrix for Highly Efficient Magnetic Wave Adsorption
Source: Nanomicro Lett. 2022 Apr 6;14:96. doi: 10.1007/s40820-022-00830-8 (PMC8986902; doi:10.1007/s40820-022-00830-8)
Supplement: Supplementary file 1 — Supplementary file1 (PDF 3617 KB) [file 40820_2022_830_MOESM1_ESM.pdf]

Supporting Information for

Ultrahigh Density of Atomic CoFe-Electron Synergy in Noncontinuous Carbon Matrix for Highly Efficient Magnetic Wave Adsorption

Wenhuan Huang[1, *], Qiang Qiu[1], Xiufang Yang[1], Shouwei Zuo[1], Jianan Bai[1], Huabin Zhang[2, *], Ke Pei[3], and Renchao Che[3, *]

[1]Key Laboratory of Chemical Additives for China National Light Industry, College of Chemistry and Chemical Engineering, Shaanxi University of Science and Technology, Xi'an 710021, P. R. China

[2]KAUST Catalysis Center, King Abdullah University of Science and Technology, 23955-6900, Thuwal, Kingdom of Saudi Arabia

[3]Laboratory of Advanced Materials, Shanghai Key Lab of Molecular Catalysis and Innovative Materials, Fudan University, Shanghai 200438, P. R. China

*Corresponding authors. E-mail: huangwenhuan@sust.edu.cn (Wenhuan Huang), huabin.zhang@kaust.edu.sa (Huabin Zhang), rcche@fudan.edu.cn (Renchao Che)

S1 Experimental Section

S1.1 Synthesis of MET

The synthesis of MET is according to the literature [S1]. $ZnCl_2$ (5.0 g) was dissolved in a mixture of ethanol (50 mL), water (75 mL), ammonium hydroxide (25%-28%, 20 mL) and N, N-dimethylformamide (DMF, 50 mL), and then kept stirring for 10 min. After that, 1H-1,2,3-triazole (6.26 mL) was slowly dropped in the solution during stirring. After a 24-hours stirring at room temperature, the white product of MET was generated and filtered out. After three times washes by ethanol, the product was dried at 80 °C for 8 h with a yield of 90%.

S1.2 Synthesis of CoFe@MET, Fe@MET, and Co@MET

For synthesizing CoFe@MET, $Co(CH_3COO)_2{\cdot}6H_2O$ (0.72 g) and $FeCl_2$ (0.49 g) in a molar ratio of 1:1 were first dissolved in 200 mL methanol as a metal solution. Then, MET (2.0 g) powder was immersed into above solution, and then stirred at room temperature for 6 h. The pink powder was filtered out and washed by ethanol for three times. After the vacuum drying at 60 °C for 8h, the CoFe@MET was collected with a yield of 62%.

The Fe@MET and Co@MET were synthesized, as contrast samples, for investigating the internal Co-Fe synergistic effect and microwave absorbing mechanism. The synthesis of reseda Fe@MET (yield of 67%) and pink Co@MET (yield of 63%) were obtained in the similar way with CoFe@MET, except form the metal solutions are instead of $FeCl_2$ (0.98 g $FeCl_2$ in 200 mL methanol) and $Co(CH_3COO)_2{\cdot}6H_2O$ (1.44 g $Co(CH_3COO)_2{\cdot}6H_2O$ in 200 mL methanol) solutions.

S1.3 Synthesis of and CoFe@PCS, Fe@PCS, Co@PCS

The as-prepared 2.0 g CoFe@MET was put into the ceramic boat and then placed in the programmed tube furnace. It was heated up to 900 °C at a heating rate of 5 °C min^{-1} under the nitrogen atmosphere. After that, the furnace was kept at 900 °C for two hours and then naturally cooled to room temperature. The ultra-light black powder of CoFe@PCS (0.16g) was successfully synthesized. The Fe@PCS and Co@PCS were

synthesized at the same condition by using Fe@MET and Co@MET as precursors.

S2 Characterizations

D8 DAVANCI X-ray powder diffractometer equipped with graphite monochromatized Cu Kα radiation (λ = 0.1542 nm) was used to record powder X-ray diffraction (PXRD) patterns in the 2θ range of 5°-80° with a scanning rate of 1 ^{o}/min. The Brunauer-Emmett-Teller (BET) method was employed to calculate the specific surface area through nitrogen adsorption and desorption at 77 K by ASAP 2020 sorption system. Scanning electron microscopy (SEM) images were collected by a Hitachi S4800 apparatus with an acceleration voltage of 2 kV. The transmission electron microscopy (TEM) images were recorded on JEM-2100F, JEM-2010HR, and FEI Talos F200X, working at an accelerating voltage of 200 kV and X-ray energy-dispersive spectroscopy (EDS) was taken on a JEM-2010HR-Vantage typed energy spectrometer. X-ray photoelectron spectroscopy (XPS) was implemented on Thermo ESCA Lab250XI. Raman spectroscopy of the samples was obtained by a Renishaw in Via Raman Microscope. The electromagnetic parameters were analyzed using a HP8753D vector network analyzer in the frequency range of 2-18 GHz. The measured samples were dispersed in paraffin homogeneously with a sample-to-paraffin weight ratio of 3:17, and then the mixture was pressed into a toroidal shape with an inner diameter of 2.0 mm and an outer diameter of 7.0 mm. The conductivity of the samples (1×1 cm^2) was performed through a ST2253 four-probe resistance meter. The hysteresis loop of the materials was tested by superconducting quantum interference device MPMS(SQUID) VSM magnetometer. The absorption spectra of Mo-edge were collected in a transmission mode at room temperature using a Si (111) double crystal monochromator at the 1W1B station of Beijing Synchrotron Radiation Facility (BSRF, Beijing).

S3 Data Calculations

The reflection loss (*RL*) values of the absorbers are calculated according to transmission line theory by the following Eqs. S1-S2 [S2]:

$$R_L(dB) = 20lg \left| \frac{Z_{in} - Z_0}{Z_{in} + Z_0} \right| \quad (S1)$$

$$Z_{in} = Z_0 \sqrt{\frac{\mu_r}{\varepsilon_r}} \tanh\left[j\left(\frac{2\pi f d}{c}\right)\sqrt{\mu_r \varepsilon_r}\right] \quad (S2)$$

Where Z_0 is the characteristic impedance of free space, Z_{in} is the normalized input impedance of absorber, ε_r and μ_r are the relative complex permittivity and permeability, *d* is the layer thickness, *c* is the speed of light in free space and *f* is the frequency.

SRL is calculated based on RL value, considering the filler loading amount and the layer thickness [S3].

$$SR_L = R_L/(\text{filler loading} \times \text{thickness}) \quad (S3)$$

Where filler loading is the wt % in test ring.

The attenuation coefficient (α) [S4]:

$$\alpha = \frac{\sqrt{2}\pi f}{C} \sqrt{(\mu''\varepsilon'' - \mu'\varepsilon') + \sqrt{(\mu''\varepsilon'' - \mu'\varepsilon')^2 + (\mu'\varepsilon'' + \mu''\varepsilon')^2}} \quad (S4)$$

Cole–Cole semicircle model (Eq. S5) [S5]:

$$(\varepsilon' - \frac{\varepsilon_{s+}\varepsilon_\infty}{2})^2 + (\varepsilon'')^2 = (\frac{\varepsilon_{s-}\varepsilon_\infty}{2})^2 \quad (S5)$$

Each semicircle in the ε′–ε″ curve stands for a polarization relaxation process. The ε_s and ε_∞ represent the static dielectric constant, the dielectric constant at infinite frequency, respectively. The high number of semicircles means the strong dipole polarization process.

Debye relaxation correction formula (Eqs. S6-S7) [S6]:

$$\varepsilon_r = \varepsilon_{r\infty} + \frac{\varepsilon_{rs} - \varepsilon_{r\infty}}{1+(i\omega\tau)^{1-A}} \quad (0 < A < 1) \tag{S6}$$

$$\varepsilon_r' = \varepsilon_{r\infty} + (\varepsilon_{rs} - \varepsilon_{r\infty}) \frac{1+(\omega\tau)^{(1-A)} sin\frac{\pi A}{2}}{1+2(\omega\tau)^{1-A} sin\frac{\pi A}{2} + (\omega\tau)^{2(1-A)}} \tag{S7}$$

ε_p'' and ε_c'' are the dielectric loss contributed by polarization relaxation and charge transport, respectively, which can be obtained according to Debye theory (Eqs. S8-S10).

$$\varepsilon" = \frac{\varepsilon_s - \varepsilon_\infty}{1+(2\pi f)^2\tau^2}\omega\tau + \frac{\sigma}{2\pi f \varepsilon_0} = \varepsilon_p'' + \varepsilon_c'' \tag{S8}$$

$$\varepsilon_c'' = \frac{\sigma}{2\pi f \varepsilon_0} \tag{S9}$$

$$\varepsilon_p'' = \frac{\varepsilon_s - \varepsilon_\infty}{1+(2\pi f)^2\tau^2}\omega\tau = \varepsilon'' - \varepsilon_c'' \tag{S10}$$

Where ε_s is the relative permittivity at static, and ε_∞ is at "infinite" high frequency. σ is the conductivity, Conductivity is a parameter used to describe the difficulty of charge flow in matter [S7].

$$(C_0 = \mu'(\mu'')^{-2}(f\,)^{-1} = 2\Pi\mu_0 d^2\sigma/3) \tag{S11}$$

S4 Supplementary Results and Disscussion

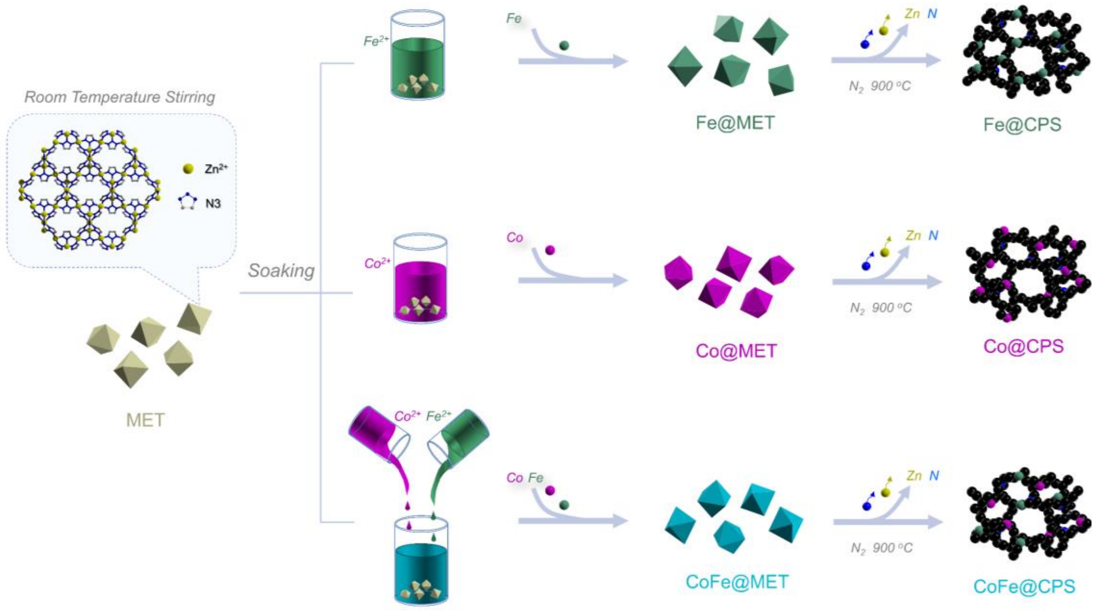

Fig. S1 The scheme of the synthesis routes for all the samples in this work

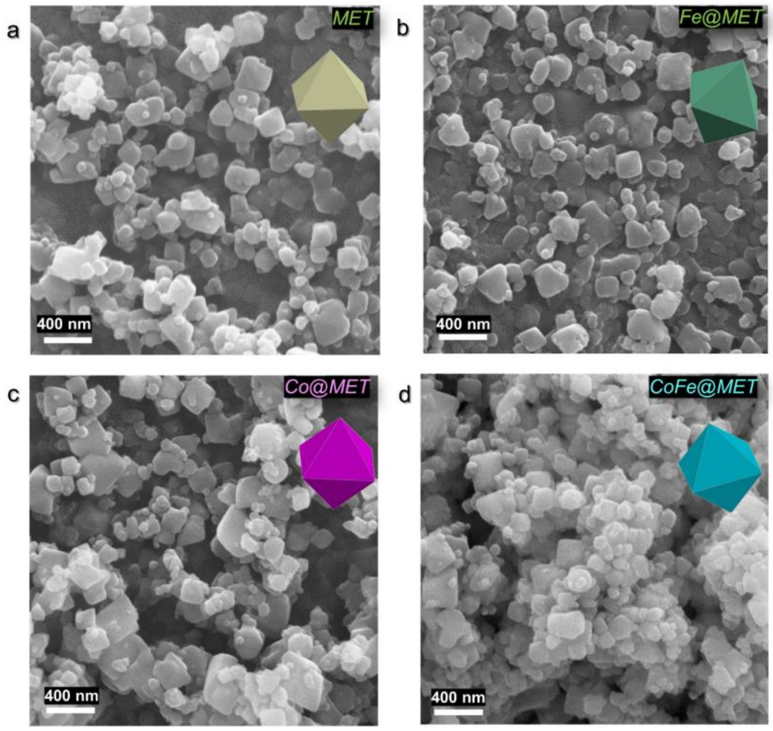

Fig. S2 SEM of (**a**) MET, (**b**) Fe@MET, (**c**) Co@MET and (**d**) CoFe@MET

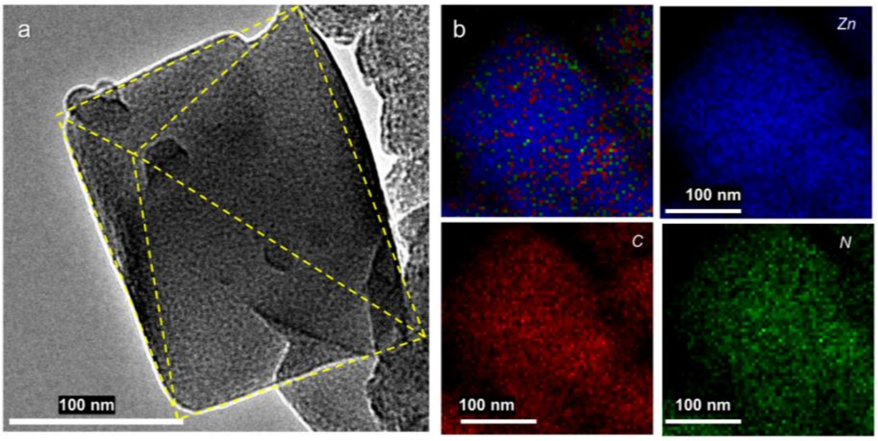

Fig. S3 TEM (**a**) and the TEM-EDS mapping (**b**) of MET

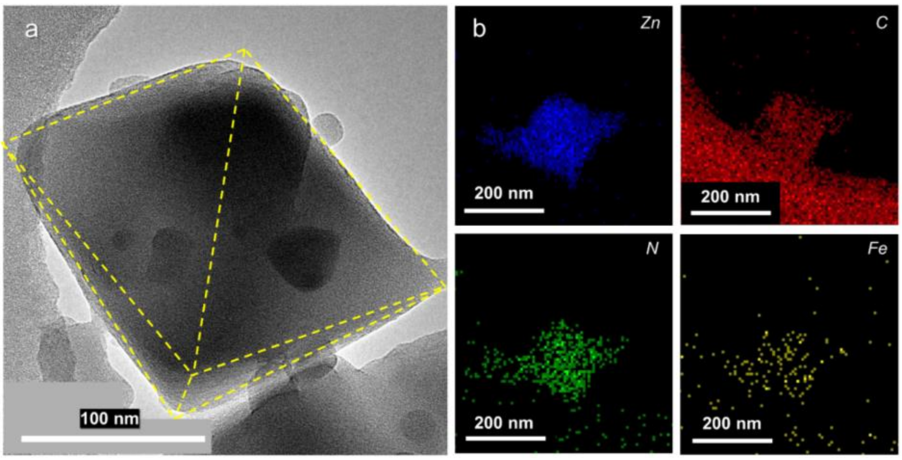

Fig. S4 TEM (**a**) and EDS mapping (**b**) of Fe@MET

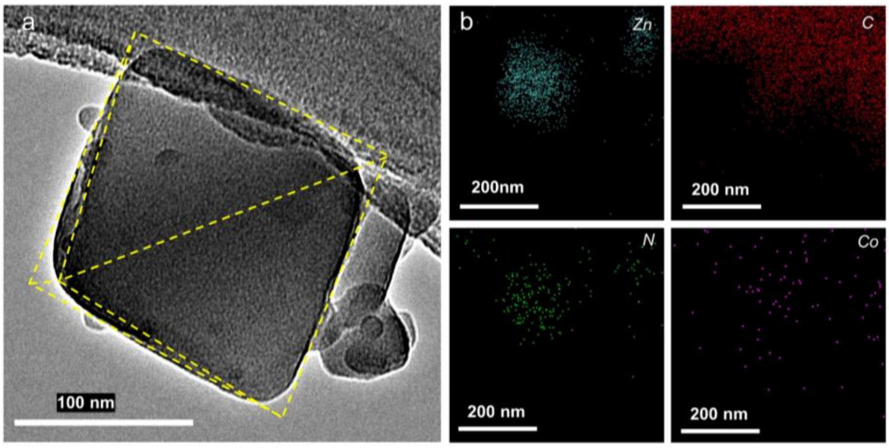

Fig. S5 TEM (**a**) and EDS mapping (**b**) of Co@MET

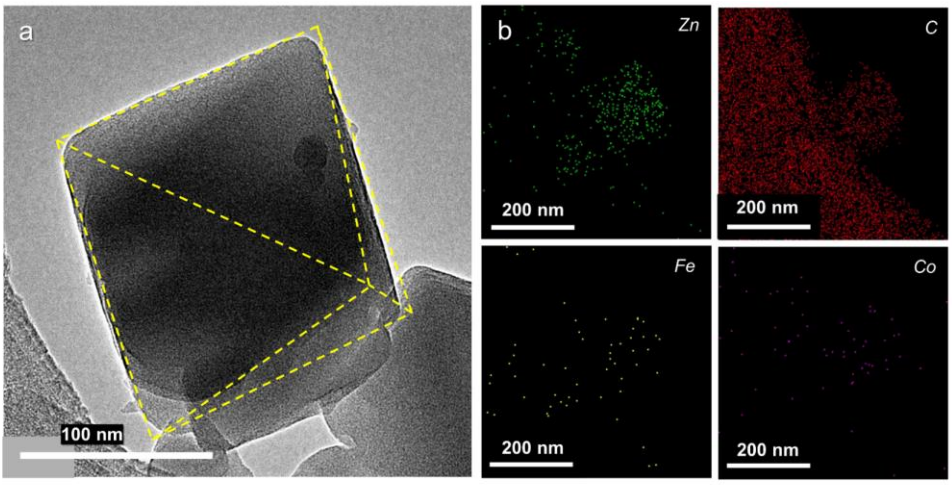

Fig. S6 TEM (**a**) and EDS mapping (**b**) of CoFe@MET

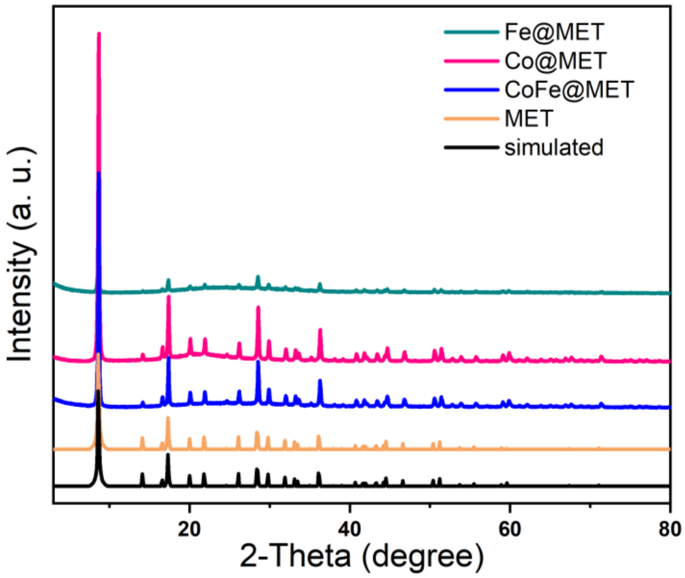

Fig. S7 PXRD results of synthesized MET, Fe@MET, Co@MET, CoFe@MET and the simulated curves of MET crystals (CCDC: 837471)

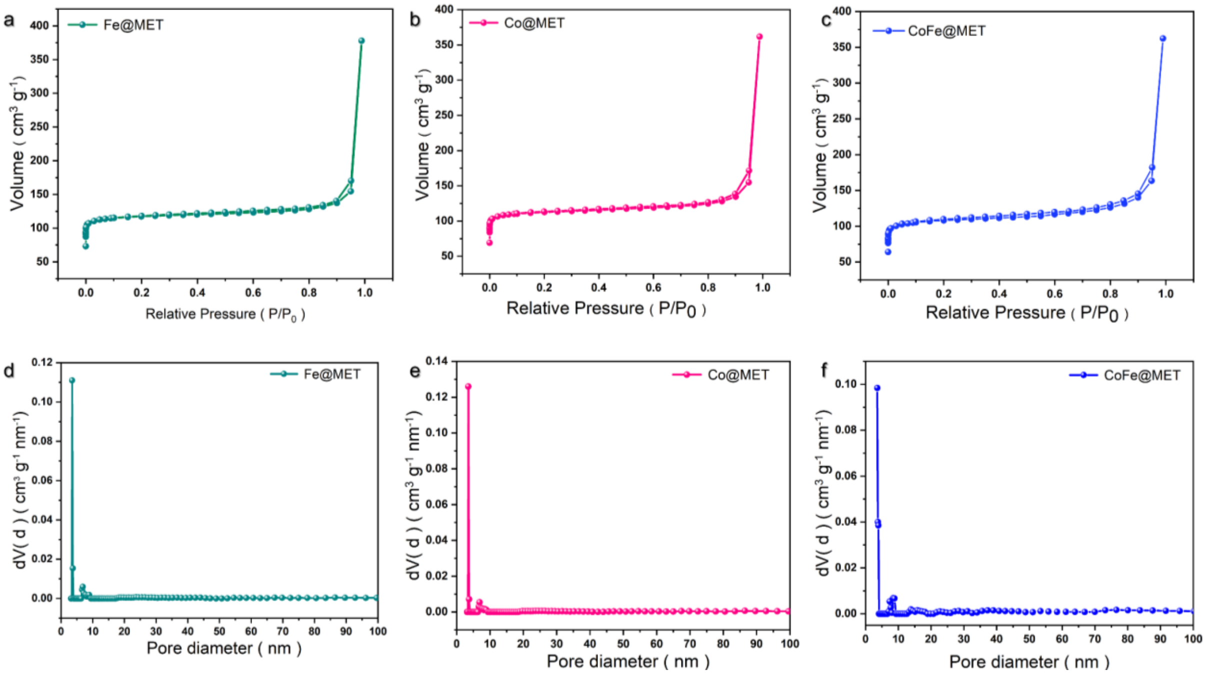

Fig. S8 (**a-c)** N_2 sorption isotherms (77 K) for Fe@MET (**a**), Co@MET (**b**), and CoFe@MET (**c**), and (**d-f**) Pore diameter distribution curves for Fe@MET (**d**), Co@MET (**e**), and CoFe@MET (**f**)

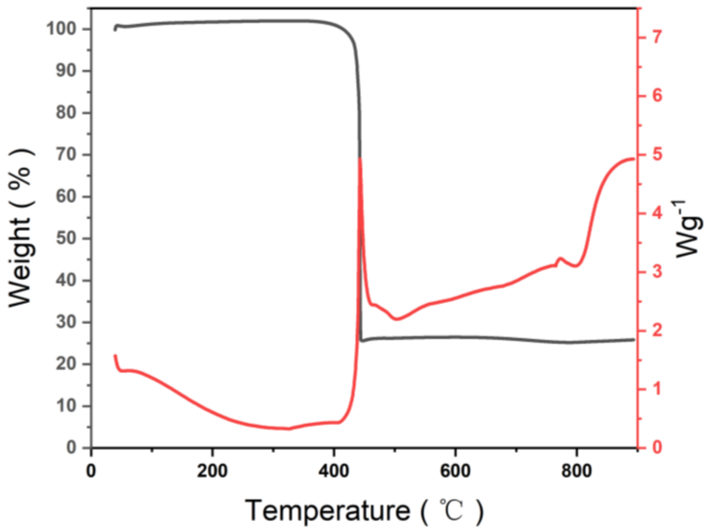

Fig. S9 The DSC and TGA curves of CoFe@MET under nitrogen atmosphere at the range of Room temperature to 900 °C

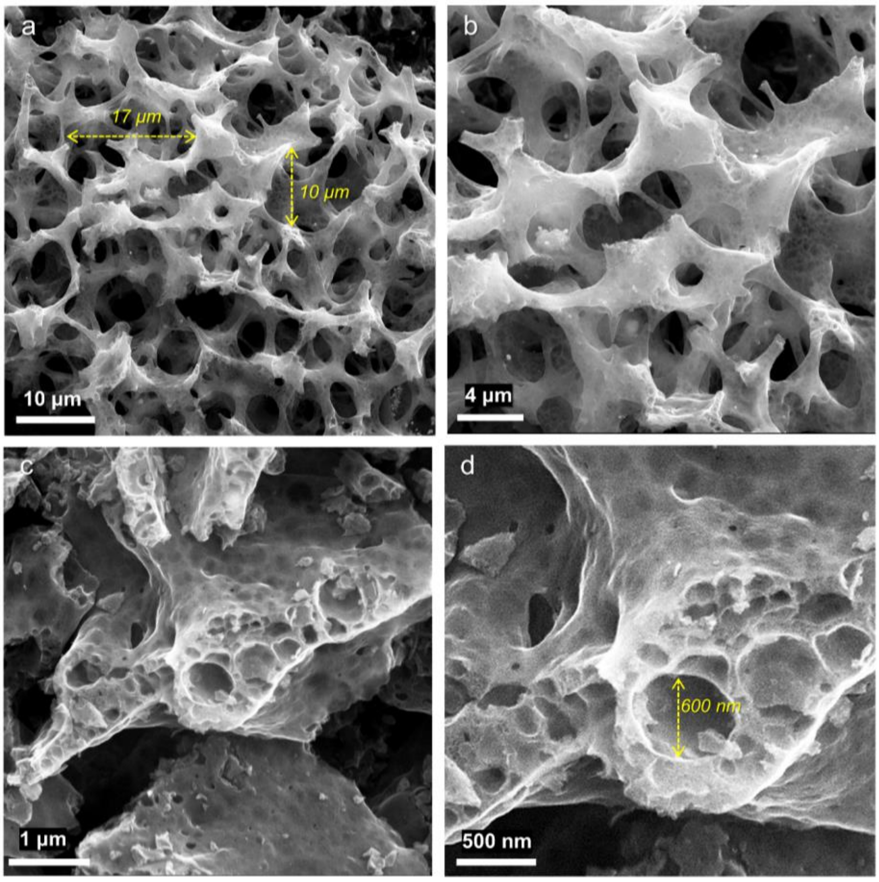

Fig. S10 SEM (**a-d**) of Fe@PCS

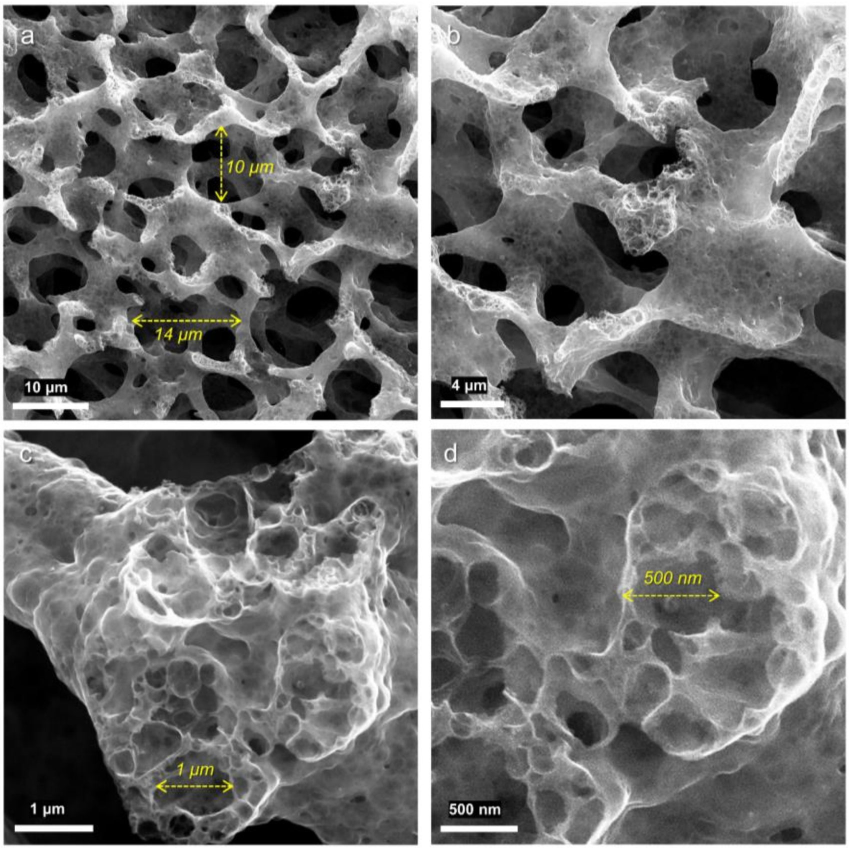

Fig. S11 SEM (**a-d**) of Co@PCS

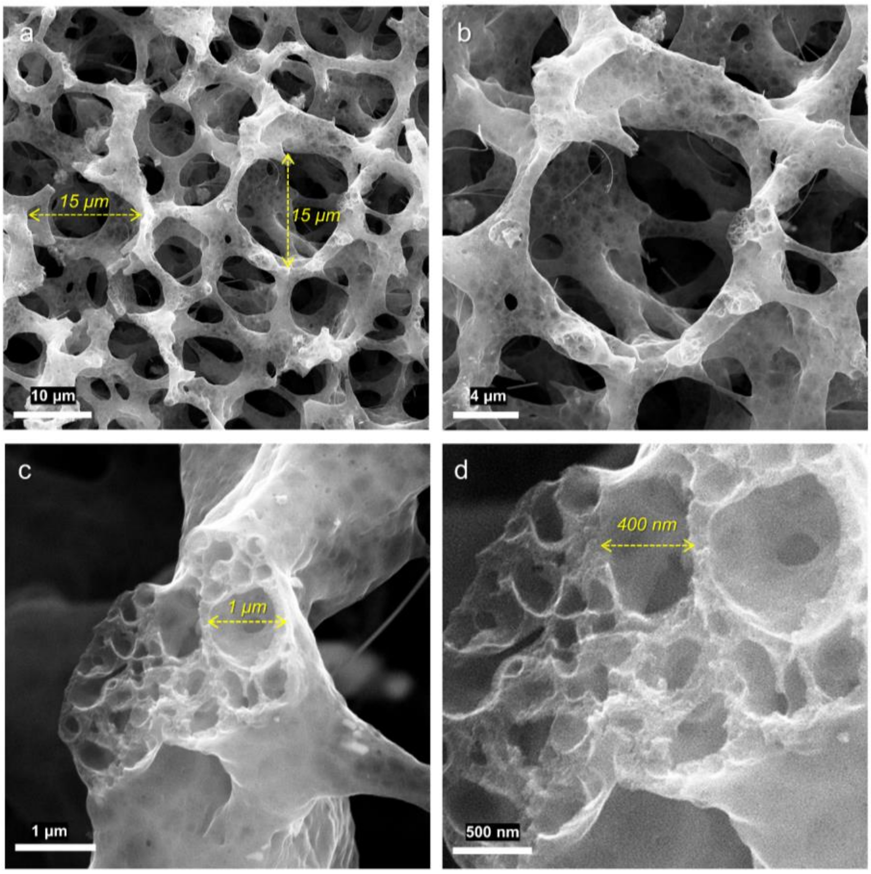

Fig. S12 SEM (**a-d**) of CoFe@PCS

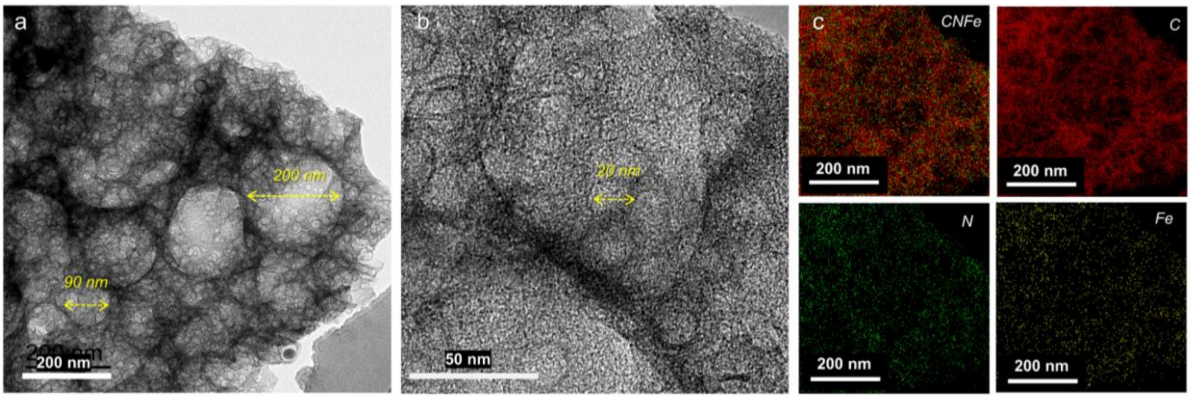

Fig. S13 TEM (**a-b**) and mapping (**c**) of Fe@PCS

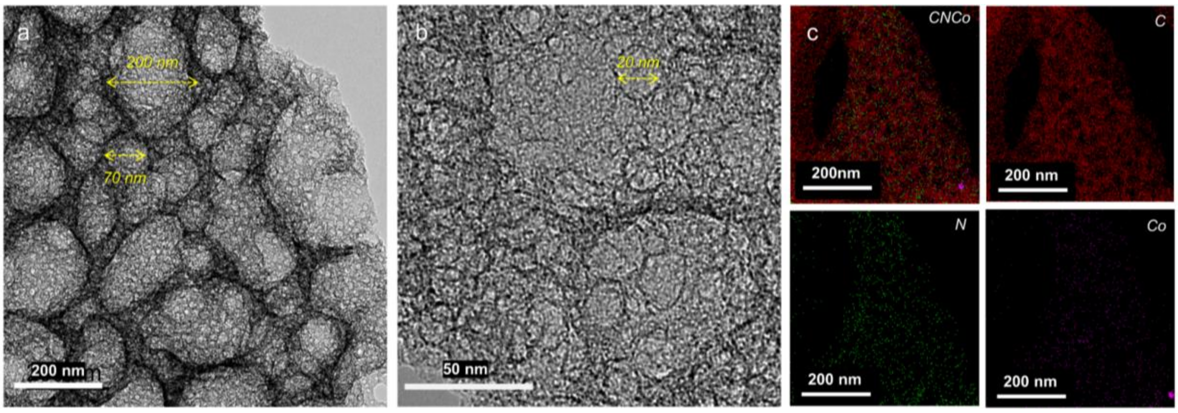

Fig. S14 TEM (**a-b**) and EDS mapping (**c**) of Co@PCS

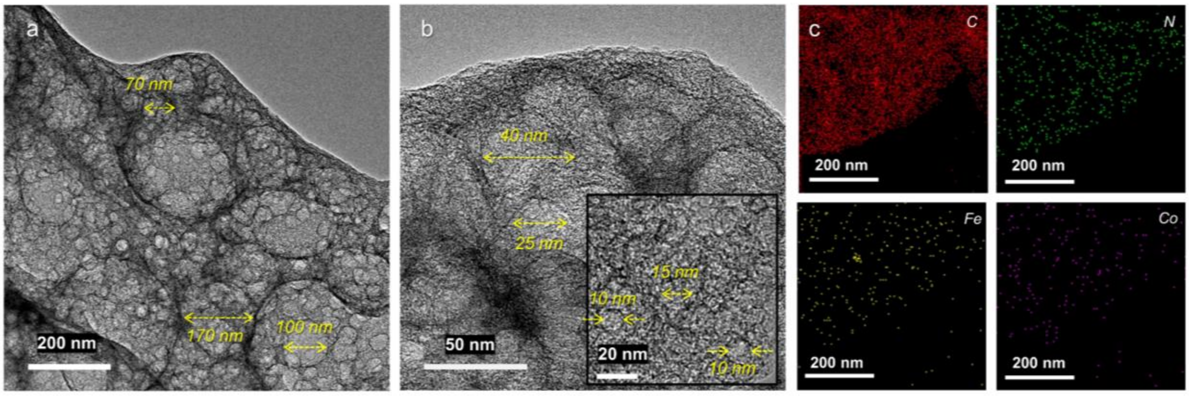

Fig. S15 TEM (**a-b**) and EDS mapping (**c**) of CoFe@PCS

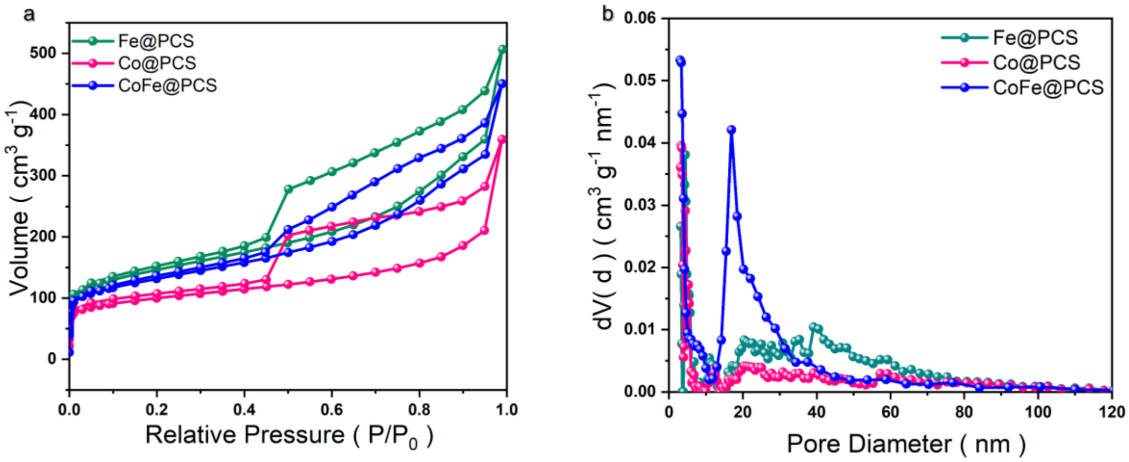

Fig. S16 (**a**) N_2 sorption isotherms (77 K) for Fe@PCS, Co@PCS, CoFe@PCS. (**b**) Pore diameter distribution curves for Fe@PCS, Co@PCS, CoFe@PCS

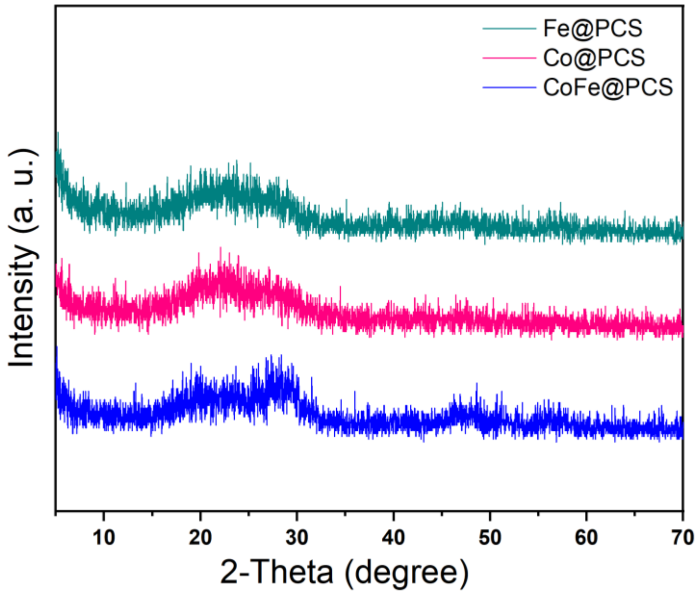

Fig. S17 PXRD of Fe@PCS, Co@PCS, and CoFe@PCS

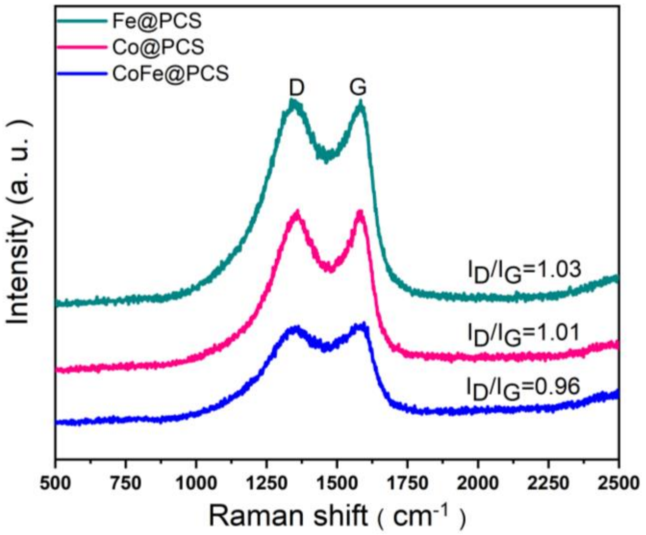

Fig. S18. Raman spectra for Fe@PCS, Co@PCS, CoFe@PCS

Table S1 ICP-OES results of of Fe@PCS, Co@PCS and CoFe@PCS

	**Fe@PCS**	**Co@PCS**	**CoFe@PCS**
**Co**	-	0.602	0.344
**Fe**	0.672	-	0.316

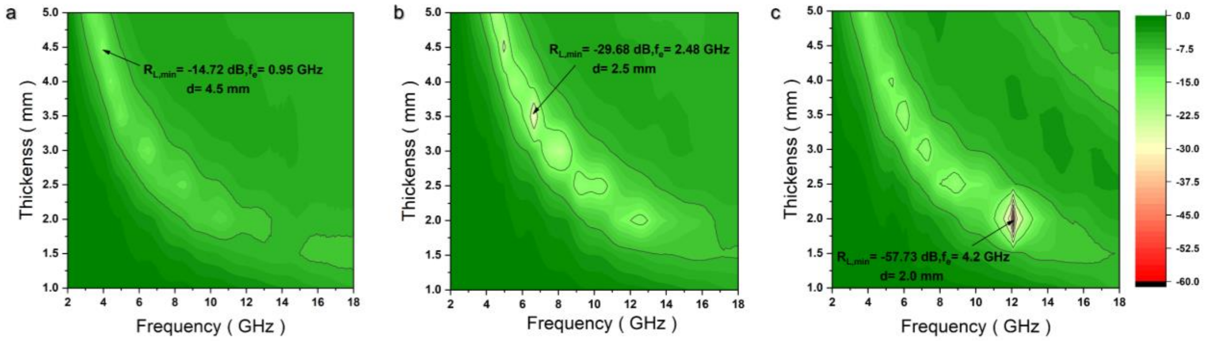

Fig. S19 2D contour map of RL for Fe@PCS (**a**), Co@PCS(**b**), CoFe@PCS(**c**)

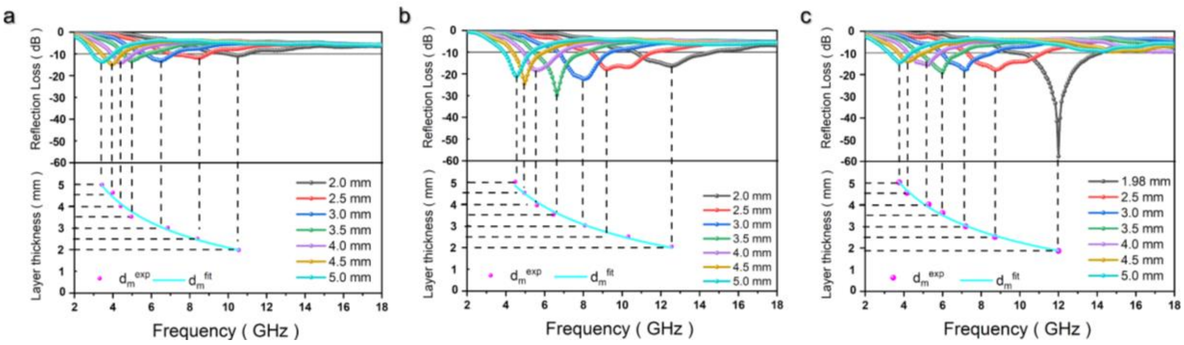

Fig. S20 The best of RL values and the effective frequency bandwidth (RL < -10 dB) of Fe@PCS (**a**), Co@PCS (**b**), CoFe@PCS (**c**).The experimental and theoretical fitted dm values of Fe@PCS (**d**), Co@PCS (**e**), CoFe@PCS (**f**)

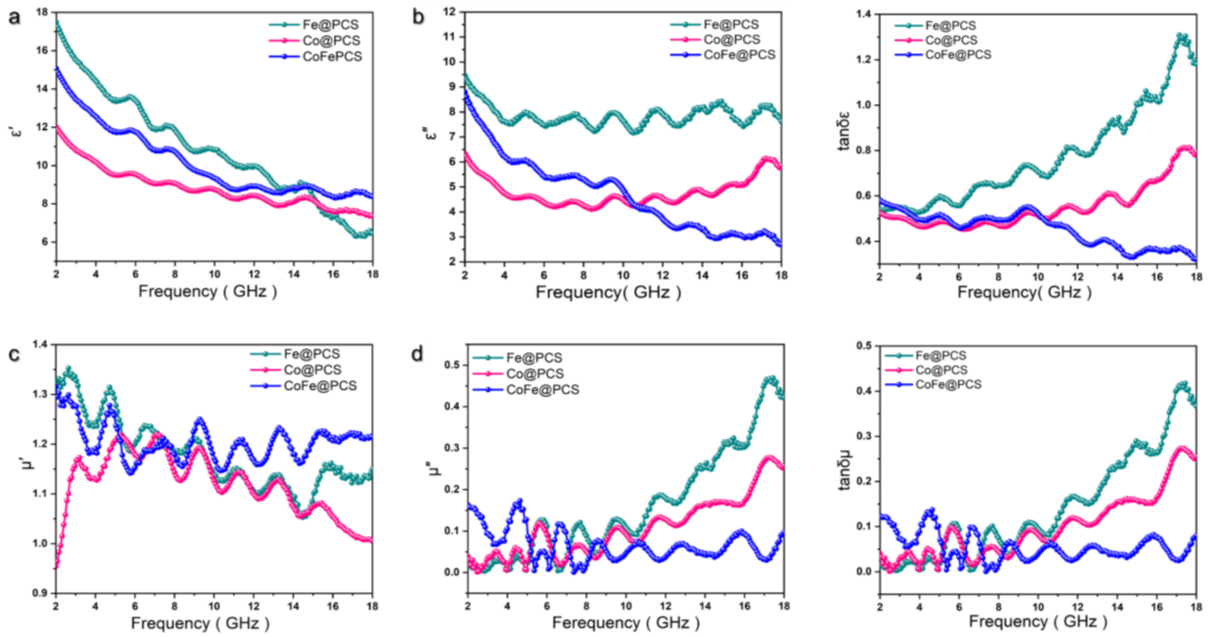

Fig. S21 The real part of permittivity (**a**), imaginary part of permittivity (**b**), dielectric loss tangent (**c**), real part of permeability (**d**), imaginary part of permeability (**e**) and magnetic loss tangent of Fe@PCS, Co@PCS and CoFe@PCS

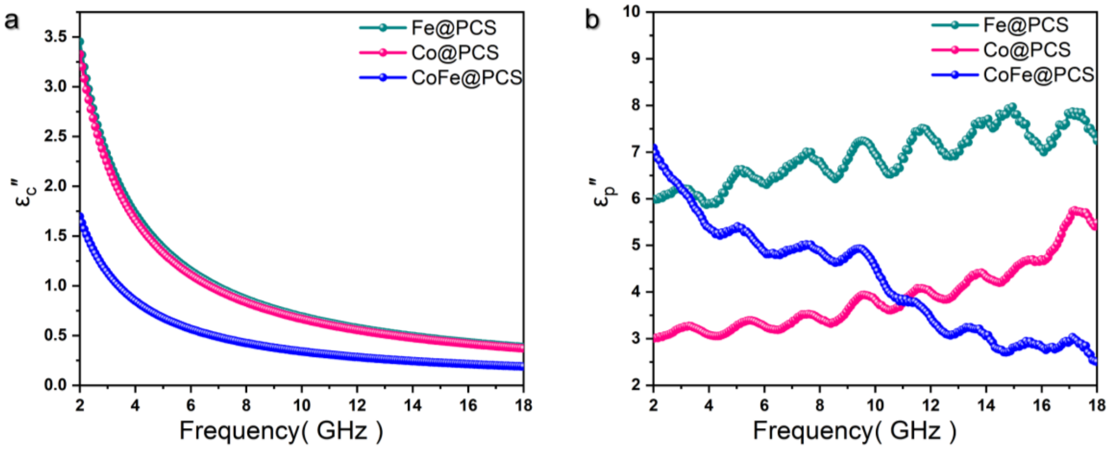

Fig. S22 The calculated ε_p'' (**a**) and ε_c'' (**b**) of Fe@PCS, Co@PCS and CoFe@PCS

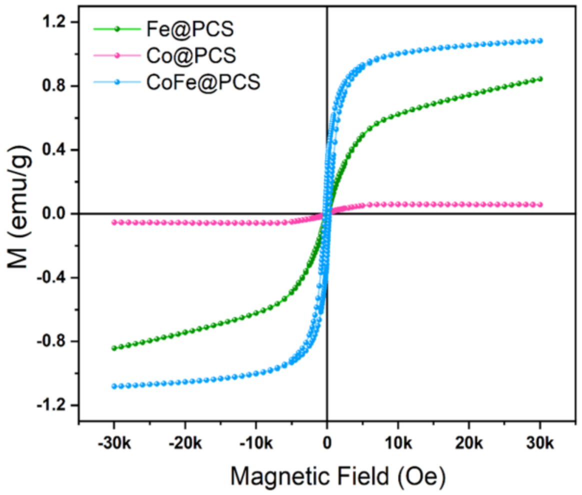

Fig. S23 The magnetic hysteresis loop of Fe@PCS, Co@PCS and CoFe@PCS

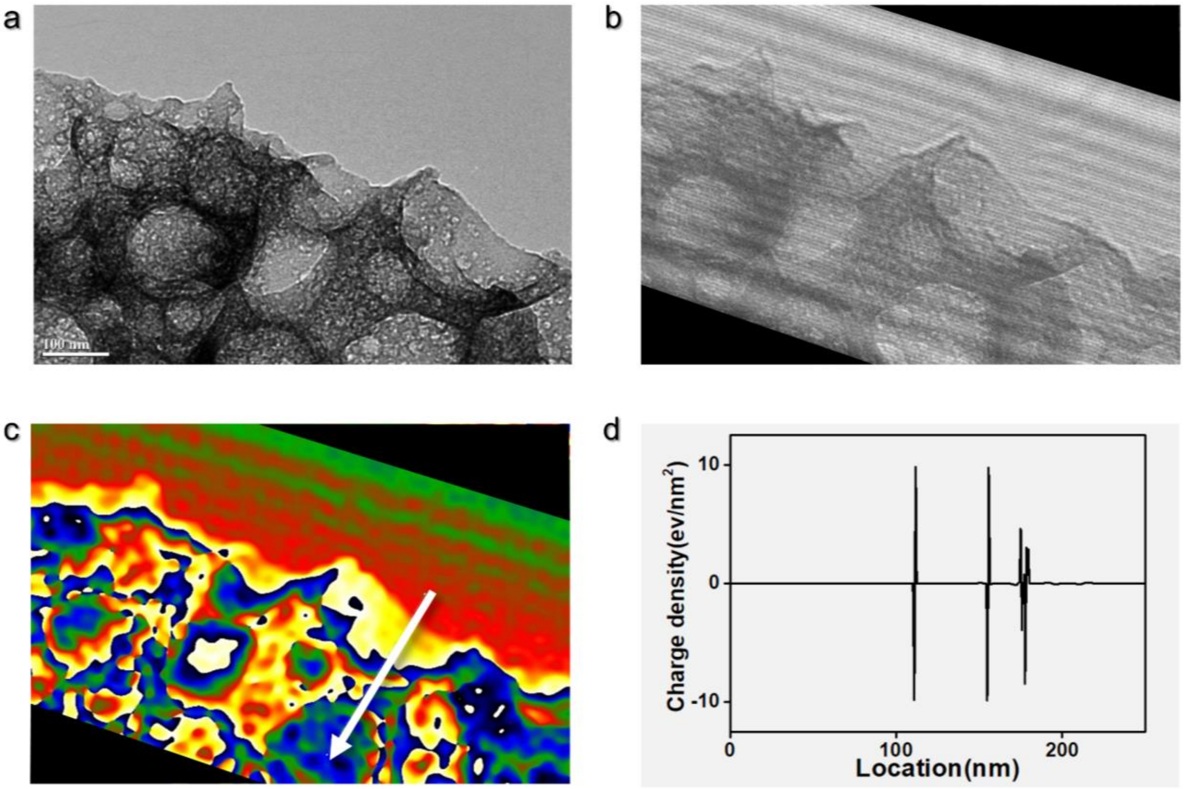

Fig. S24 HR-TEM and its corresponding hologram images (**a-b**), charge density map (**c**), the profile of charge density in the region along the white arrow (**d**) of Fe@PCS

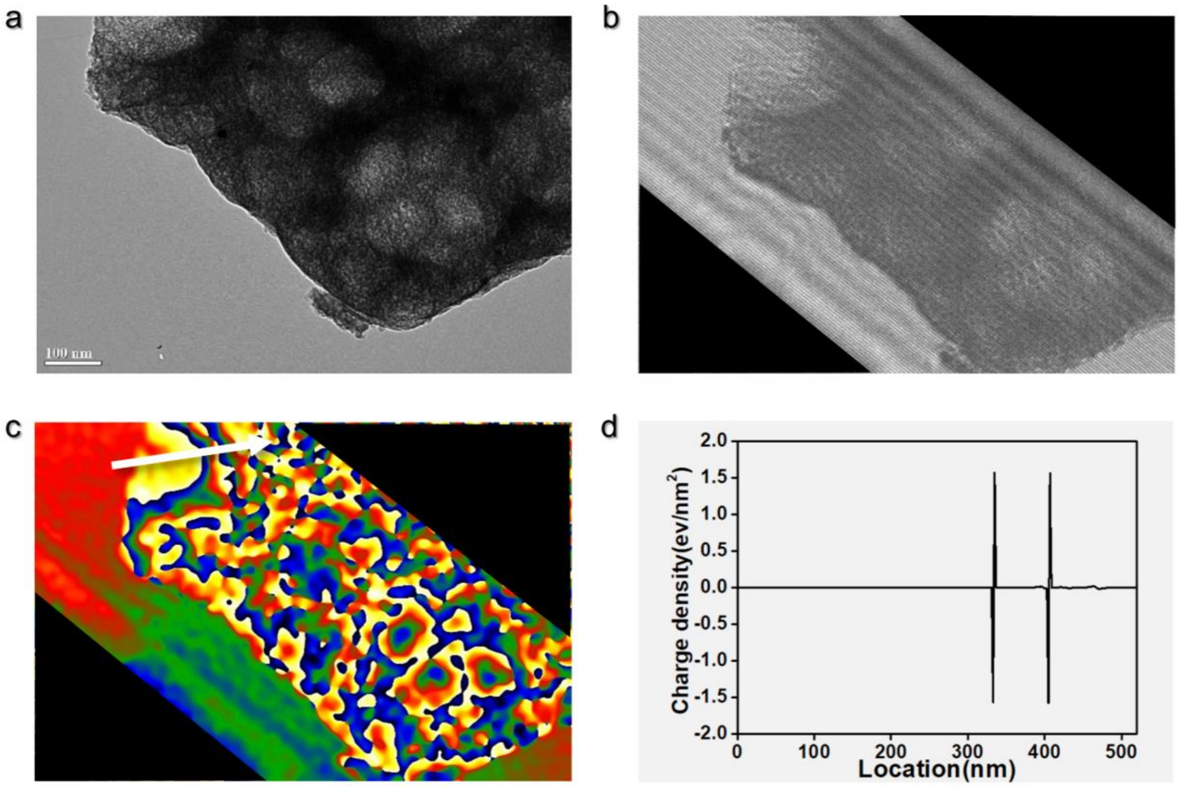

Fig. S25 HR-TEM and its corresponding hologram images (**a-b**), charge density map (**c**), the profile of charge density in the region along the white arrow (**d**) of Co@PCS

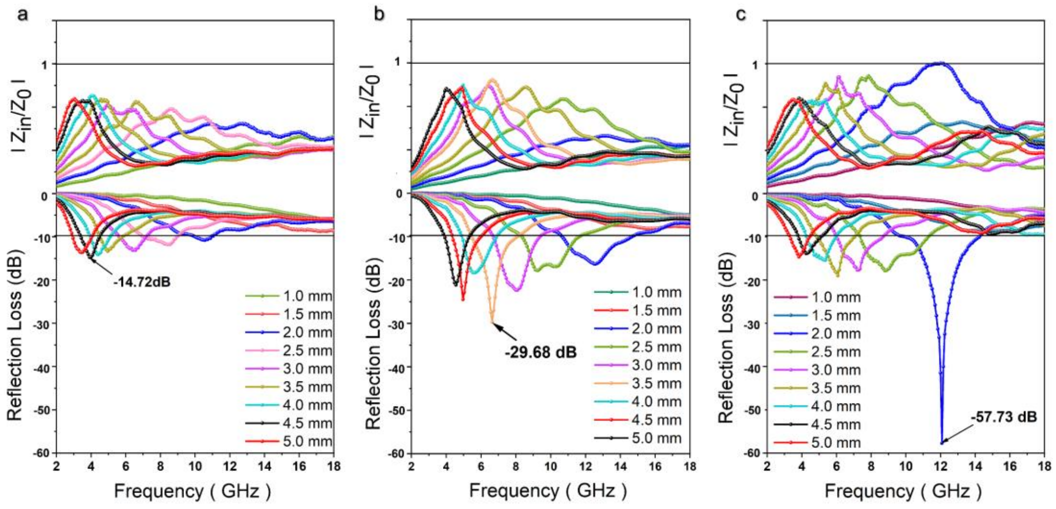

Fig. S26 $|Z_{in}/Z_0|$ and RL plots of Fe@PCS,Co@PCS and CoFe@PCS

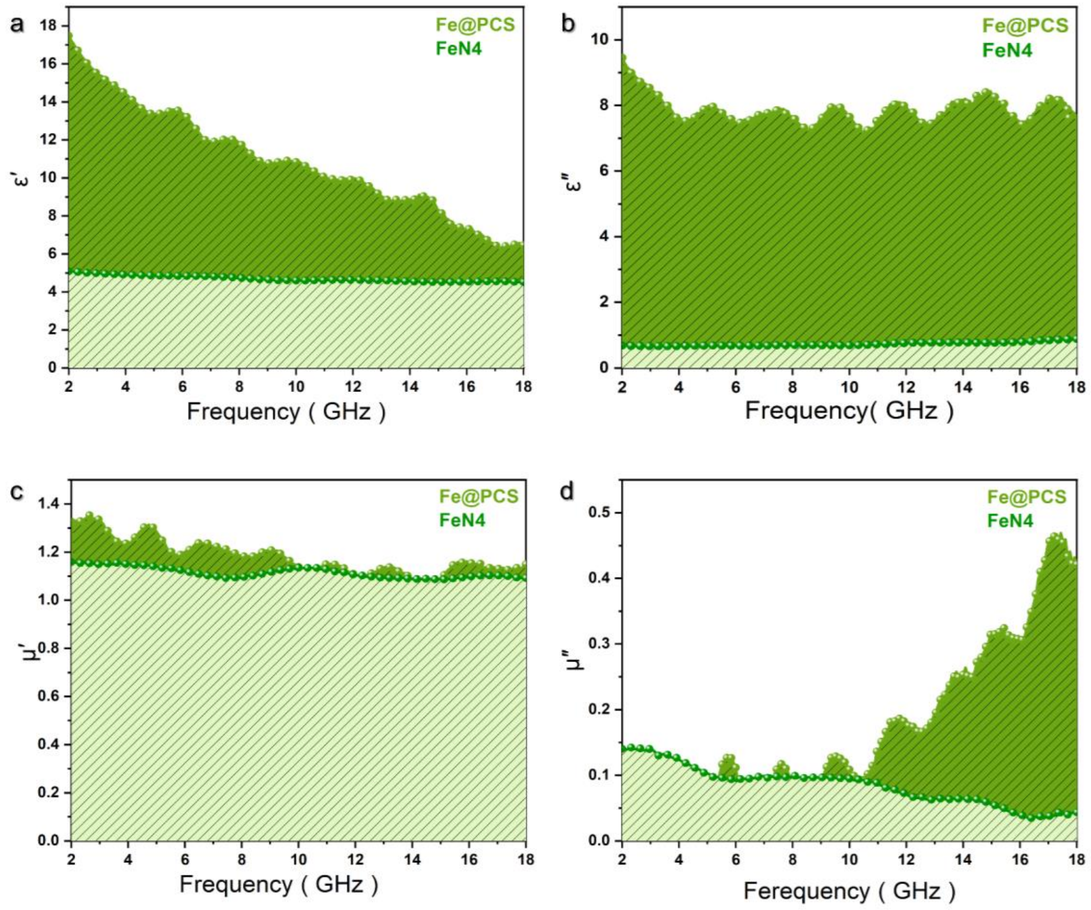

Fig. S27 ε', ε'', μ' and μ'' contribution of Fe-N_4 in Fe@PCS

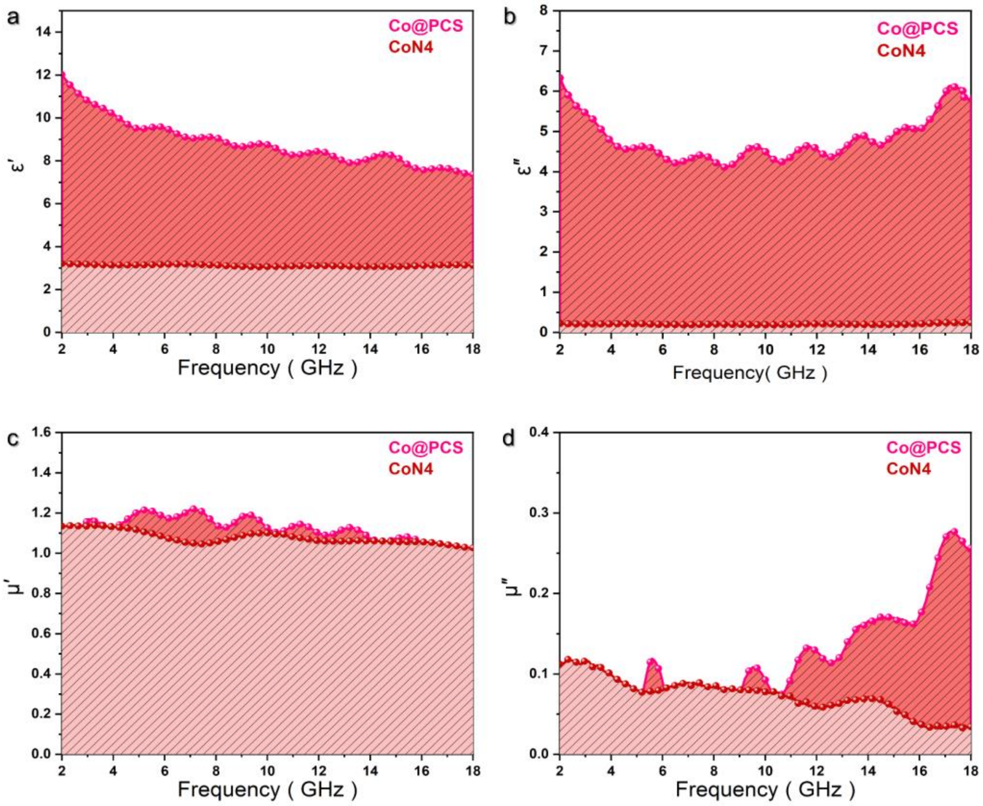

Fig. S28 ε', ε'', μ' and μ'' contribution of Co-N_4 in Co@PCS

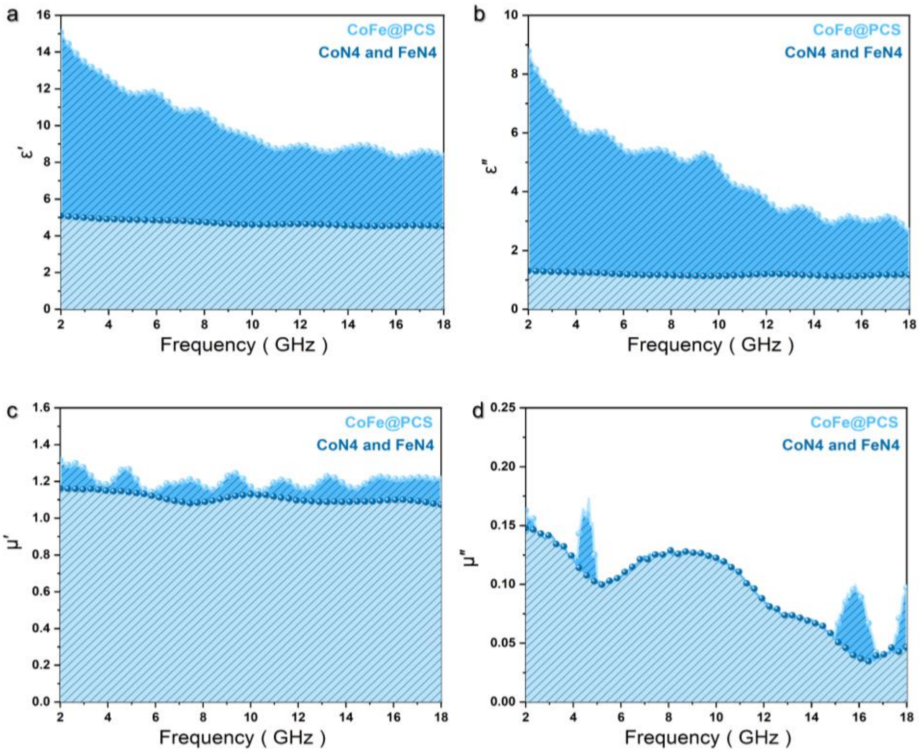

Fig. S29 ε', ε'', μ' and μ'' contribution of Fe/Co-N_4 in CoFe@PCS

Table S2 Comparison of microwave absorption performance of CoFe@PCS with other reported CoFe-based carbon EW absorbers

**Samples**	***R*$_L$ (dB)**	**Thickness (mm)**	**Bandwidth (GHz)**	**Loading (wt%)**	**S*R*$_L$ (dB mg^{-1} mm^{-1})**	**Refs.**
CNT-CoFe@C-900	−40.00	3.0	5.62	10%	-133	[S8]
$Co_{0.8}Fe_{2.2}O_4$/rGO	−51.20	2.1	5.7	30%	-81	[S9]
CFs@H-Fe_3O_4/CoFe	−40.85	3.5	2.14	30%	-39	[S10]
$Co_{0.2}Fe_{2.8}O_4$	−43.45	3.0	5.58	30%	-48	[S11]
CoFe@N-CNT/rGO	−33.2	2.0	3.8	50%	-33	[S12]
$Co_{15}Fe_{85}$@C/RGO-2	−33.4	2.5	9.2	30%	-44	[S13]
Fe–Co/NC/rGO	−43.36	2.5	9.29	30%	-57	[S14]
MWCNTs/FeCoNi@C	−36	2.0	4.0	30%	-60	[S15]
Fe-Co/NPC/RGO	−52.9	2.5	3.1	30%	-70	[S16]
$CoFe_2O_4$/FeCo	-54.3	1.2	15.2	50%	-90	[S17]
CoFe@C@MnO_2	-64	1.3	9.2	30%	-164	[S18]
ZnO/FeNiMo	-28	1.5	4.54	30%	-61	[S19]
rGO/$CoFe_2O_4$	-56.3	1.4	3.4	30%	-134	[S20]
Co_{1-x}NixFe_2O_4	-37.66	2.1	2.64	30%	-59	[S21]
$CoFe_{0.26}$@Co@C	-62.5	1.5	14.7	30%	-138	[S22]
CoFe2/BTO-1 HNFs	-82.4	1.2	5	50%	-137	[S23]
FeCoNi-MOF-74	-64.75	2.1	8.08	38%	-81	[S24]
$Ba_{0.8}Dy_{0.2}Co_2Fe_{16}O_{27}$	-15	1.5	7	30%	-33	[S25]
Ni(Co/Zn/Cu)Fe_2O_4/SiC@SiO_2	-32.76	3.0	2.1	30%	-36	[S26]
CoFe@PCS-900	−57.7	2.0	4.24	15%	-192	This work

Supplementary References

[S1] F. Gándara, F.J. Uribe-Romo, D.K. Britt, H. Furukawa, L. Lei et al., Porous, conductive metal-triazolates and their structural elucidation by the charge-flipping method. Chem. Eur. J. **18**(34), 10595-10601 (2012). https://doi.org/10.1002/chem.201103433

[S2] E. Cui, F. Pan, Z. Xiang, Z. Liu, L. Yu et al., Engineering dielectric loss of FeCo/polyvinylpyrrolidone core-shell nanochains@graphene oxide composites with excellent microwave absorbing properties. Adv. Eng. Mater. **23**(1), 2000827 (2020). https://doi.org/10.1002/adem.202000827

[S3] J. Wang, L. Liu, S. Jiao, K. Ma, J. Lv et al., Hierarchical carbon fiber@MXene@MoS_2 core-sheath synergistic microstructure for tunable and efficient microwave absorption. Adv. Funct. Mater. **30**(45), 2002595 (2020). https://doi.org/10.1002/adfm.202002595

[S4] X. Gao, X. Wu, J. Qiu, High electromagnetic waves absorbing performance of a multilayer-like structure absorber containing activated carbon hollow porous fibers–carbon nanotubes and Fe_3O_4 nanoparticles. Adv. Electron. Mater. **4**(5), 1700565 (2018). https://doi.org/10.1002/aelm.201700565

[S5] P. Liu, Y. Zhang, J. Yan, Y. Huang, L. Xia et al., Synthesis of lightweight N-doped graphene foams with open reticular structure for high-efficiency electromagnetic wave absorption. Chem. Eng. J. **368**, 285-298 (2019). https://doi.org/10.1016/j.cej.2019.02.193

[S6] F. Pan, Z. Liu, B. Deng, Y. Dong, X. Zhu et al., Lotus leaf-derived gradient hierarchical porous C/MoS_2 morphology genetic composites with wideband and tunable electromagnetic absorption performance. Nano-Micro Lett. **13**, 43 (2021). https://doi.org/10.1007/s40820-020-00568-1

[S7] C. Wu, Z. Chen, M. Wang, X. Cao, Y. Zhang et al., Confining tiny MoO_2 clusters into reduced graphene oxide for highly efficient low frequency microwave absorption. Small **16**(30), 2001686 (2020). https://doi.org/10.1002/smll.202001686

[S8] Y. Wang, H. Wang, J, Ye, L. Shi, X. Feng, Magnetic CoFe alloy@C nanocomposites derived from ZnCo-MOF for electromagnetic wave absorption. Chem. Eng. J. **383**, 123096 (2019). https://doi.org/10.1016/j.cej.2019.123096

[S9] W. Shen, B. Ren, K. Cai, Y. Song, W. Wang, Synthesis of nonstoichiometric $Co_{0.8}Fe_{2.2}O_4$/reduced graphene oxide (rGO) nanocomposites and their excellent electromagnetic wave absorption property. J. Alloys Compd. **774**, 997-1008 (2019). https://doi.org/10.1016/j.jallcom.2018.09.361

[S10] P. Yin, L. Zhang, J. Wang, X. Feng, J. Dai et al., Facile preparation of cotton-derived carbon fibers loaded with hollow Fe_3O_4 and CoFe NPs for significant low-frequency electromagnetic absorption. Powder Technol. **380**, 134-142 (2021). https://doi.org/10.1016/j.powtec.2020.11.044

[S11] Y. Wang, Y. Zhang, J. Tao, W. Sun, M. Liu et al., $Co_{0.2}Fe_{2.8}O_4$/C composite nanofibers with designable 3D hierarchical architecture for high-performance electromagnetic wave absorption. Ceram. Int. **47**(16), 23275-23284 (2021). https://doi.org/10.1016/j.ceramint.2021.05.040

[S12] X. Zhang, Z. Zhao, J. Xu, X. Ouyang, C. Zhu et al., N-doped carbon nanotube arrays on reduced graphene oxide as multifunctional materials for energy devices and

absorption of electromagnetic wave. Carbon **177**, 216-225 (2021). https://doi.org/10.1016/j.carbon.2021.02.085

[S13] S. Bao, W. Tang, Z. Song, Q. Jian, Z. Jiang et al., Synthesis of sandwich-like $Co_{15}Fe_{85}$@C/RGO multicomponent composites with tunable electromagnetic parameters and microwave absorption performance. Nanoscale **12**(36), 18790-18799 (2020). https://doi.org/10.1039/D0NR04615A

[S14] S. Wang, Y. Xu, R. Fu, H. Zhu, Q. Jiao et al., Rational construction of hierarchically porous Fe–Co/N-doped carbon/rGO composites for broadband microwave absorption. Nano-Micro Lett. **11**, 76 (2019). https://doi.org/10.1007/s40820-019-0307-8

[S15] O. Mirzaee, I. Huynen, M. Zarainejad, Electromagnetic wave absorption characteristics of single and double layer absorbers based on trimetallic FeCoNi@C metal−organic framework incorporated with MWCNTs. Synth. Met. **271**, 116634 (2021). https://doi.org/10.1016/j.synthmet.2020.116634

[S16] Y. Wang, X. Gao, C. Lin, L. Shi, X. Li et al., Metal organic frameworks-derived Fe-Co nanoporous carbon/graphene composite as a high-performance electromagnetic wave absorber. J. Alloys Compd. **785**, 765-773 (2019). https://doi.org/10.1016/j.jallcom.2019.01.271

[S17] X. Su, J. Wang, X. Zhang, S. Huo, W. Chen et al., One-step preparation of $CoFe_2O_4$/FeCo/graphite nanosheets hybrid composites with tunable microwave absorption performance. Ceram. Int. **46**(8), 12353-12363 (2020). https://doi.org/10.1016/j.ceramint.2020.01.286

[S18] Y. Zhang, Z. Yang, M. Li, L.Yang, J. Liu et al., Heterostructured CoFe@C@MnO_2 nanocubes for efficient microwave absorption. Chem. Eng. J. **382**, 123039 (2019). https://doi.org/10.1016/j.cej.2019.123039

[S19] L. Zheng, H, Zhu, M. Li, Preparation and application in electromagnetic absorption of ZnO/FeNiMo composite. Rare Met. **33**(5), 573-577 (2013). https://doi.org/10.1007/s12598-013-0146-6

[S20] H. Zong, H. Yang, J. Dong, L. Ma, Y. Lin et al., Construction of a three-dimensional rGO/$CoFe_2O_4$ nanorods composite with enhanced microwave absorption performance. J. Mater. Sci. Mater. Electron. **31**(21),18590-18604 (2020). https://doi.org/10.1007/s10854-020-04402-7

[S21] Z. Tong, Q. Yao, J. Feng, L. Cheng, T. Chuang et al., Effects of Ni-doping on microstructure, magnetic and microwave absorption properties of $CoFe_2O_4$. Mater. Sci. Eng. B **268**, 115092 (2021). https://doi.org/10.1016/j.mseb.2021.115092

[S22] B. Wang, W. Ruan, C. Mu, A. Nie, F. Wen et al., Direct one-step synthesis of CoFex@Co@C hybrids derived from a metal organic framework for a lightweight and high-performance microwave absorber. Nanotechnology **31**(9), 95703 (2020). https://doi.org/10.1088/1361-6528/ab5620

[S23] G. Guan, G. Gao, J. Xiang, J. Yang, L. Gong et al., $CoFe_2$/$BaTiO_3$ hybrid nanofibers for microwave absorption. ACS Appl. Nano Mater. **3**(8), 8424-8437 (2020). https://doi.org/10.1021/acsanm.0c01855

[S24] J. Quyang, Z. He, Y. Zhang, H. Yang, Q. Zha, Trimetallic FeCoNi@C nanocomposite hollow spheres derived from metal–organic frameworks with superior electromagnetic wave absorption ability. ACS Appl. Nano Mater. Interfaces **11**(42), 39304-39314 (2019). https://doi.org/10.1021/acsami.9b11430

[S25] L. Wang, X. Hang, J. Zhang, H. Wang, Q. Zhang et al., Microstructure and microwave electromagnetic properties of Dy^{3+}-doped W-type hexaferrites. Rare Met. **30**(5),505-509 (2021). https://doi.org/10.1007/s12598-011-0420-4

[S26] P. Wang, P. Liu, S. Ye, Preparation and microwave absorption properties of Ni(Co/Zn/Cu)Fe_2O_4/SiC@SiO_2 composites. Rare Met. **38**(1), 59-63 (2016). https://doi.org/10.1007/s12598-016-0752-1
